# Computational Characterization of the DAD Photoisomerization: Functionalization, Protonation, and Solvation Effects

**DOI:** 10.1021/acs.jpcb.4c05179

**Published:** 2024-11-16

**Authors:** Lucía López-Pacios, Juan J. Nogueira, Lara Martínez-Fernández

**Affiliations:** † Departamento de Química, Facultad de Ciencias, Universidad Autónoma de Madrid, Campus de Excelencia UAM-CSIC, Cantoblanco, 28049 Madrid, Spain; ‡ Institute for Advanced Research in Chemistry (IAdChem), Universidad Autónoma de Madrid, Campus de Excelencia UAM-CSIC, Cantoblanco, 28049 Madrid, Spain; § Departamento de Química Física de Materiales, Instituto de Química Física Blas Cabrera, CSIC, 28006 Madrid, Spain

## Abstract

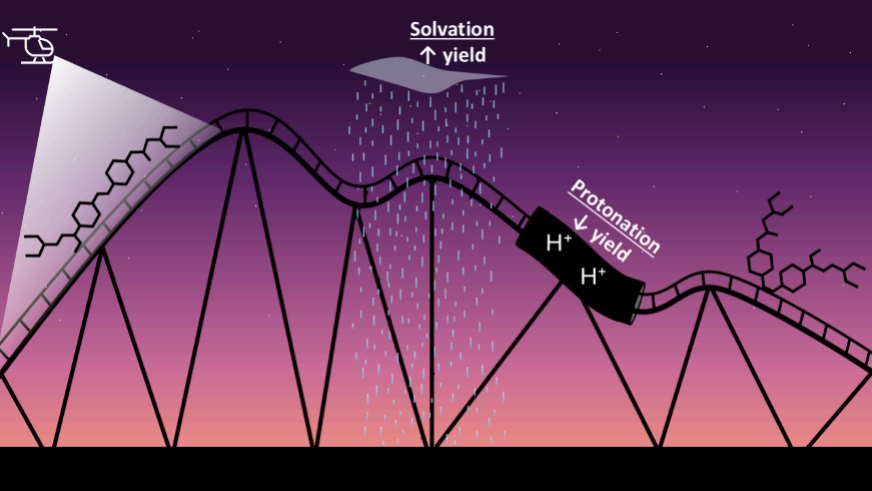

Photoswitches are becoming increasingly popular in pharmacology due to the possibility of modifying their activity with light. Hence, it is crucial to understand the photophysics of these compounds to identify promising light-activated drugs. We focused our study on DAD, an azobenzene derivative that, according to a previous experimental investigation, can restore visual function in blind mice due to *trans*-*cis* photoisomerization upon light absorption. With the present computational study, we aim to characterize the absorption spectrum of DAD, and to understand its photoisomerization mechanism by means of conformational search analysis, quantum mechanical (QM) and hybrid QM/continuum calculations, and classical molecular dynamics simulations. Moreover, we explored the effect of the derivation (DAD vs azobenzene), the protonation (DAD vs DADH_2_^2+^, the two possible protonation states) and the solvation (vacuum vs water) on the photoisomerization. Similarly to azobenzene, we showed that the photoisomerization of both protonation states of DAD begin with the population of the bright *S*_2_ state. Then, it crosses to the *S*_1_ surface and relaxes along the rotation of the azo dihedral to a *S*_1_/*S*_0_ crossing point. The latter is close to a transition state that connects the *trans* and *cis* geometries on the ground state. Finally, our results suggested that amino derivation, nonprotonation and water solvation could improve the quantum yield of the photoisomerization.

## Introduction

Photopharmacology is an emerging field aimed to tackle the toxicity and selectivity issues of conventional drugs. The use of drugs that get activated by light of a specific wavelength allows a more precise spatiotemporal control of their activity compared to conventional drugs, thus reducing their side-effects. In particular, the study of photoswitches as light-activated drugs, which undergo a reversible conformational change upon light irradiation, has become increasingly popular.^1−7^ To ensure their effectiveness and safety, the drug conformation after irradiation should be biologically active only in the desired target, whereas the other conformation, or dark conformation, needs to be inactive and nontoxic everywhere. In addition, the photoactivation wavelength should belong to the so-called therapeutic window, i.e., it should be long enough to penetrate tissues avoiding the ultraviolet (UV) region.^1−3^

**Figure 1:**
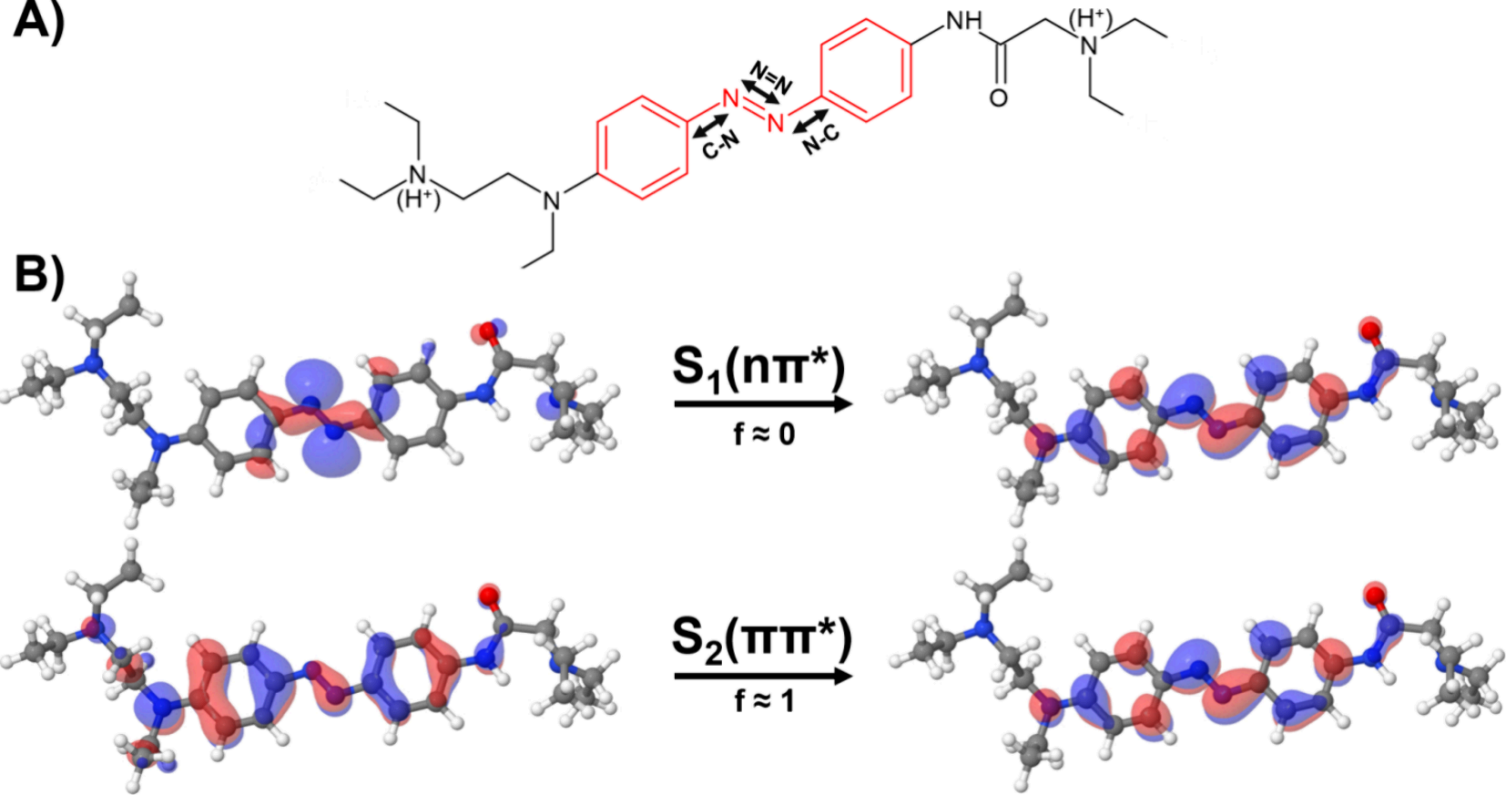
(A) Chemical structure of AB (in red) and its derivative DAD. Protonation occurs in the tertiary amino groups that are labeled with (H^+^). The C–N, *N*=N, and N–C distances of the azo moiety are also indicated. (B) Molecular orbitals and oscillator strengths (f) of the two first singlet excited states (*S*_1_ and *S*_2_) of DAD.

Azobenzene (AB) derivatives are the most widely used photoswitches to date as many of them fulfill most of the requirements previously stated. AB (Figure 1A) exists in both *cis* and *trans* conformations. The *trans* conformation is more stable than the *cis* one and, thus, the former is more abundant in the dark. The *trans*-AB absorption spectrum consists of two bands in the UV–vis region: a very strong band at λ_max_ = 316 nm corresponding to the bright ππ* *S*_2_ excitation, and a much weaker band at λ_max_ = 443 nm that is attributed to the dark nπ* *S*_1_ transition.^8^ On the other hand, the *cis*-AB absorption spectrum presents at λ_max_ = 430 nm a more intense nπ* *S*_1_ band compared to the nπ* of *trans*-AB, since the transition becomes symmetry allowed, and the brighter *S*_2_ ππ* band at λ_max_ = 280 nm.^8^

The photoisomerization of AB has been widely studied both from experimental and theoretical perspectives.^9−45^ Although its mechanism is still unclear, recent research supports that the conformational change occurs in the surface of the *S*_1_ (nπ*) excited state.^11,12,28−31^ This pathway is accessible upon direct *S*_*1*_ nπ* excitation after irradiation with long wavelength lasers, or after internal conversion from the bright *S*_2_ (ππ*) state, which is more probably populated at high enough excitation energies due to its higher oscillator strength. However, the quantum photoisomerization yield is lower following the bright *S*_2_ ππ* excitation (0.10–0.15) than when the dark *S*_1_ nπ* state is directly populated (0.20–0.28).^10,12,18,41,42^ This suggests that AB follows different isomerization mechanisms depending on the excitation wavelength and more pathways may become accessible when the photoswitch is excited to the *S*_2_ state.^9,12,16,18,28,30,43^ The latter may be due to the energy excess after the *S*_2_*/S*_1_ internal conversion and, thus, a decrease in the *S*_2_–*S*_1_ energy gap could improve the quantum yield. In some cases, the functionalization of AB results in *S*_2_ red shifting, leading to a *S*_2_–*S*_1_ energy gap decrease and a better quantum yield. Moreover, the required radiation is less harmful and penetrates deeper into the tissues.^18^

This work focuses on the study of the AB derivative diethylamino-azo-diethylamino (DAD), depicted in Figure 1A. It has been designed so that the AB moiety allows the conformational change upon irradiation, and that its substituents, not only red shift the *S*_2_ band, but also bind to the biological target: an ion channel.^46,47^ DAD has been used to restore retinal light responses and light-driven behavior to blind mice.^47^ According to this experimental work, the most abundant isomer in the dark, the *trans*-DAD, blocks a voltage-gated K^+^ channel and then, upon 454 nm irradiation, it photoisomerizes to *cis*-DAD and unblocks the channel. Then, the *cis*-DAD relaxes back to the *trans*-DAD in the dark within 200 ms. Furthermore, DAD exists in both deprotonated and protonated forms. The former state can diffuse rapidly across biological barriers due to its nonpolar nature, making it convenient to target transmembrane proteins like ion channels. Then, when the chromophore is embedded in the protein environment, both protonation states are possible depending on the p*K*_a_ and on its route of access to the ion-channel. To our knowledge, there are no theoretical studies on DAD. In this sense, we aim to provide the mechanism of the photoisomerization of DAD, as well as its comparison with AB, to understand the effect of the substituents on the quantum yield by means of quantum mechanics (QM)/continuum calculations and molecular dynamics (MD) simulations. Moreover, we aim to characterize the excited states involved in the photophysics of DAD, and to assess the effect of the solvent and the protonation state on the deactivation process following the dark *S*_1_ nπ* and the bright *S*_2_ ππ* excitations (see involved orbitals in Figure 1B). Specifically, we found the lowest *S*_2_–*S*_1_ energy gap for deprotonated DAD in water, followed by diprotonated DAD (DADH_2_^2+^) in water, DAD in vacuum, DADH_2_^2+^ in vacuum and AB in vacuum. Hence, the derivation of AB and solvation lead to a lower *S*_2_–*S*_1_ energy gap, whereas protonation increased it. In addition, low *S*_2_–*S*_1_ energy gaps probably lead to a more straightforward photoisomerization due to the absence of minima in the nπ* and ππ* surfaces.

**Figure 2:**
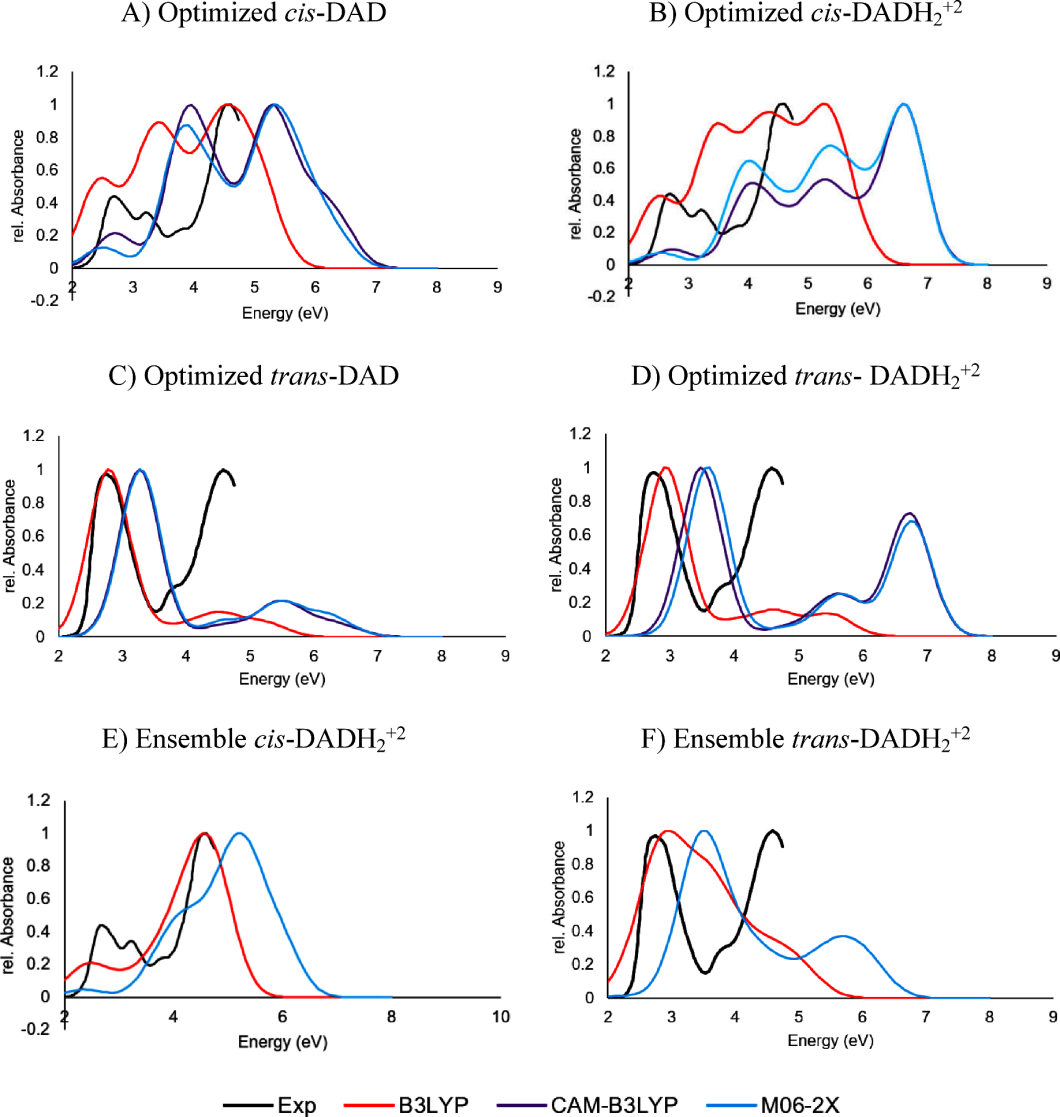
Optimized geometry vertical absorption spectra in water of the optimized structures of (A) *cis*-DAD, (B) *cis*-DADH_2_^+2^, (C) *trans*-DAD, and (D) *trans*-DADH_2_^+2^ at the B3LYP (red), CAM-B3LYP (purple), and M06-2X/cc-pVDZ (blue) levels of theory. Ensemble vertical absorption spectra in water (IEFPCM) of (E) *cis*-DADH_2_^+2^ and (F) *trans*-DADH_2_^+2^ at the B3LYP and M06-2X/cc-pVDZ levels of theory. The experimental *cis* and *trans*-DAD spectra from ref (47) are plotted in black. The absorbance intensity is represented relative to the maximum value of each spectrum.

### Computational Details

**Figure 3:**
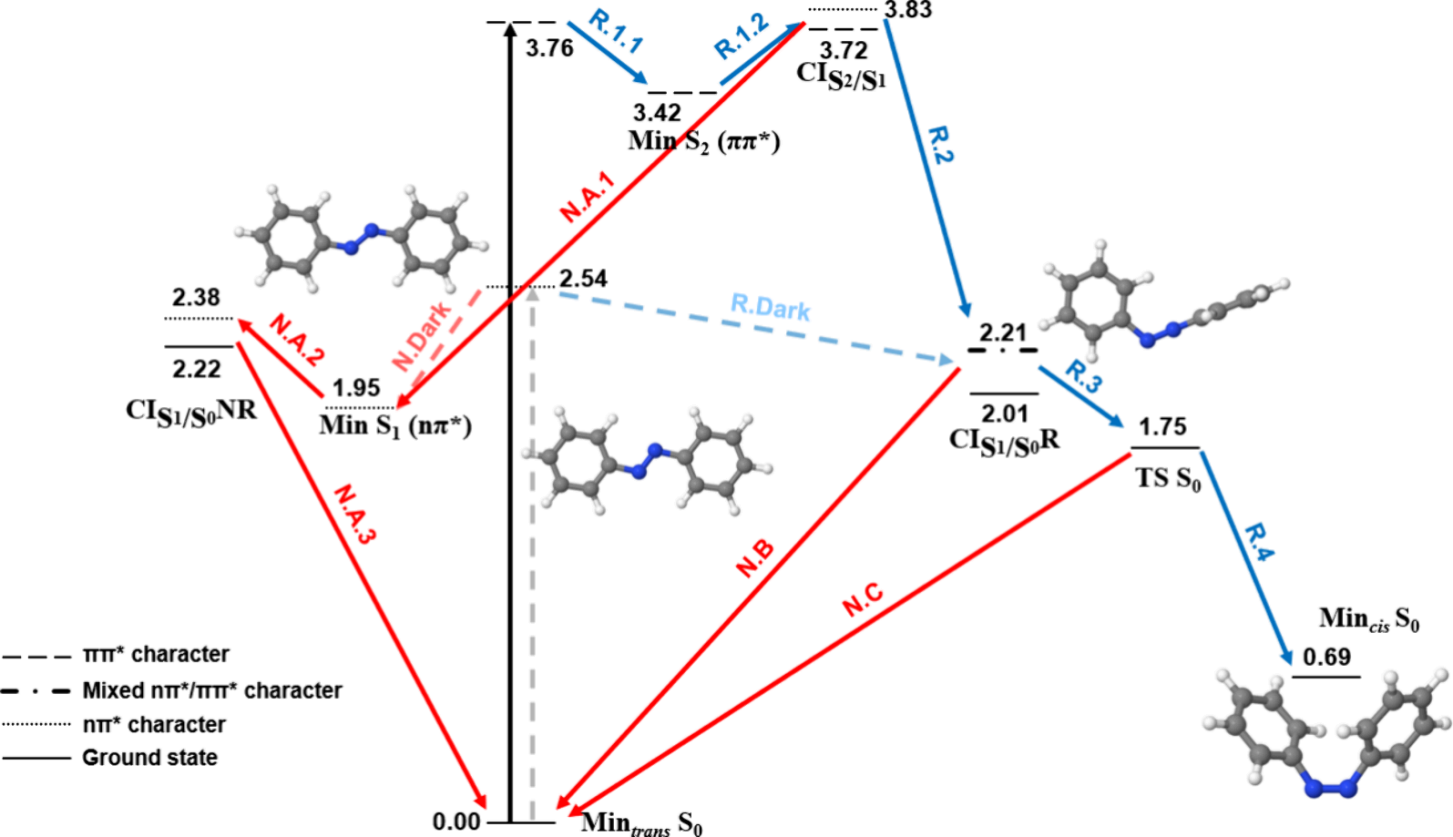
Relaxation pathways of *trans*-AB in vacuum at B3LYP/cc-pVDZ. Blue and red arrows indicate reactive (R) and nonreactive pathways (N), respectively. Continuous and dashed arrows represent the bright ππ* and dark nπ* excitations, respectively, and their corresponding relaxation pathways. Energies in eV relative to Min_*trans*_*S*_0_.

**Figure 4:**
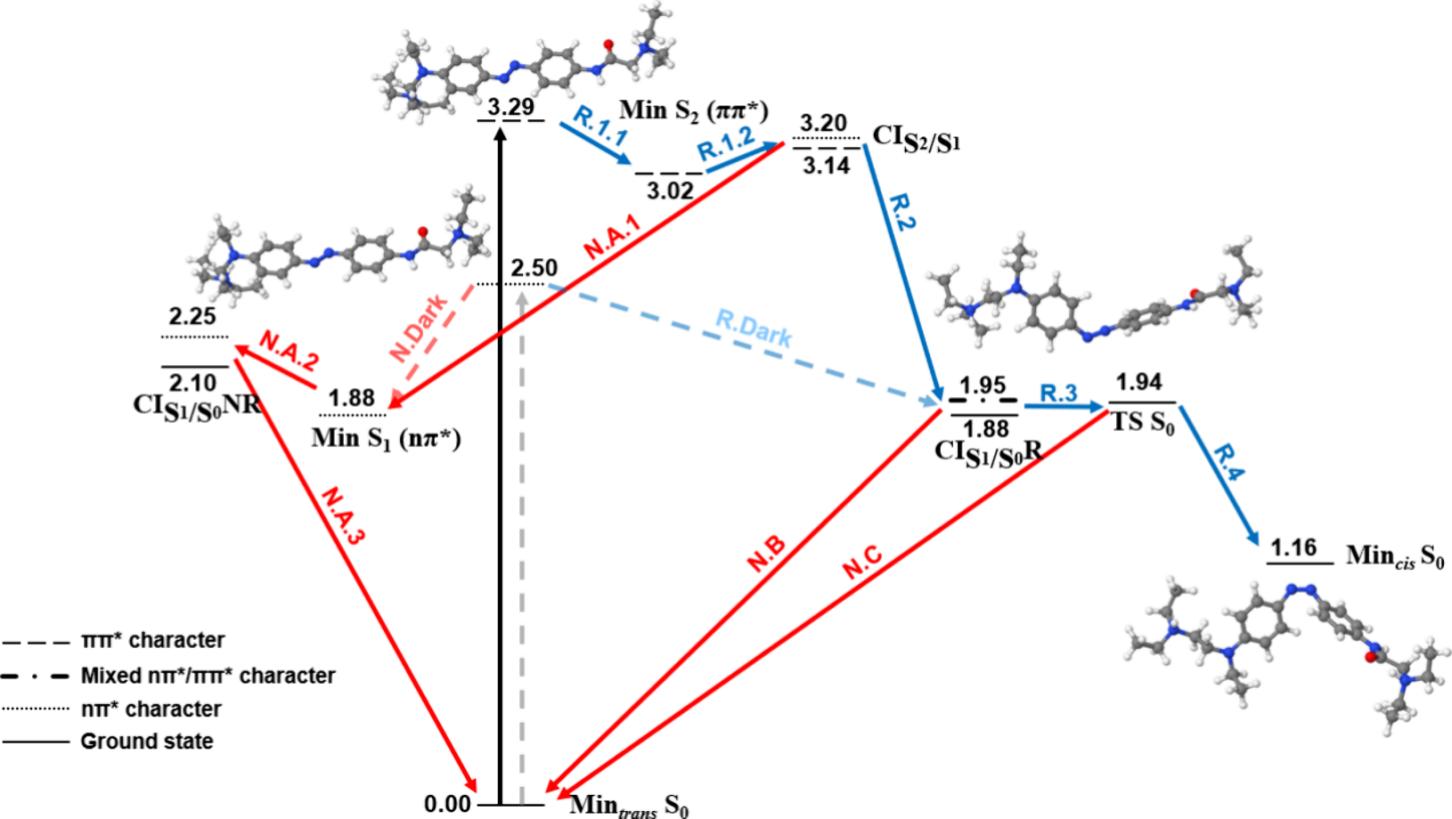
Relaxation pathways of *trans*-DADH_2_^+2^ in vacuum at B3LYP/cc-pVDZ. Blue and red arrows indicate reactive (R) and nonreactive pathways (N), respectively. Continuous and dashed arrows represent the bright ππ* and dark nπ* excitations, respectively, and their corresponding relaxation pathways. Energies in eV relative to Min_*trans*_*S*_0_.

**Figure 5:**
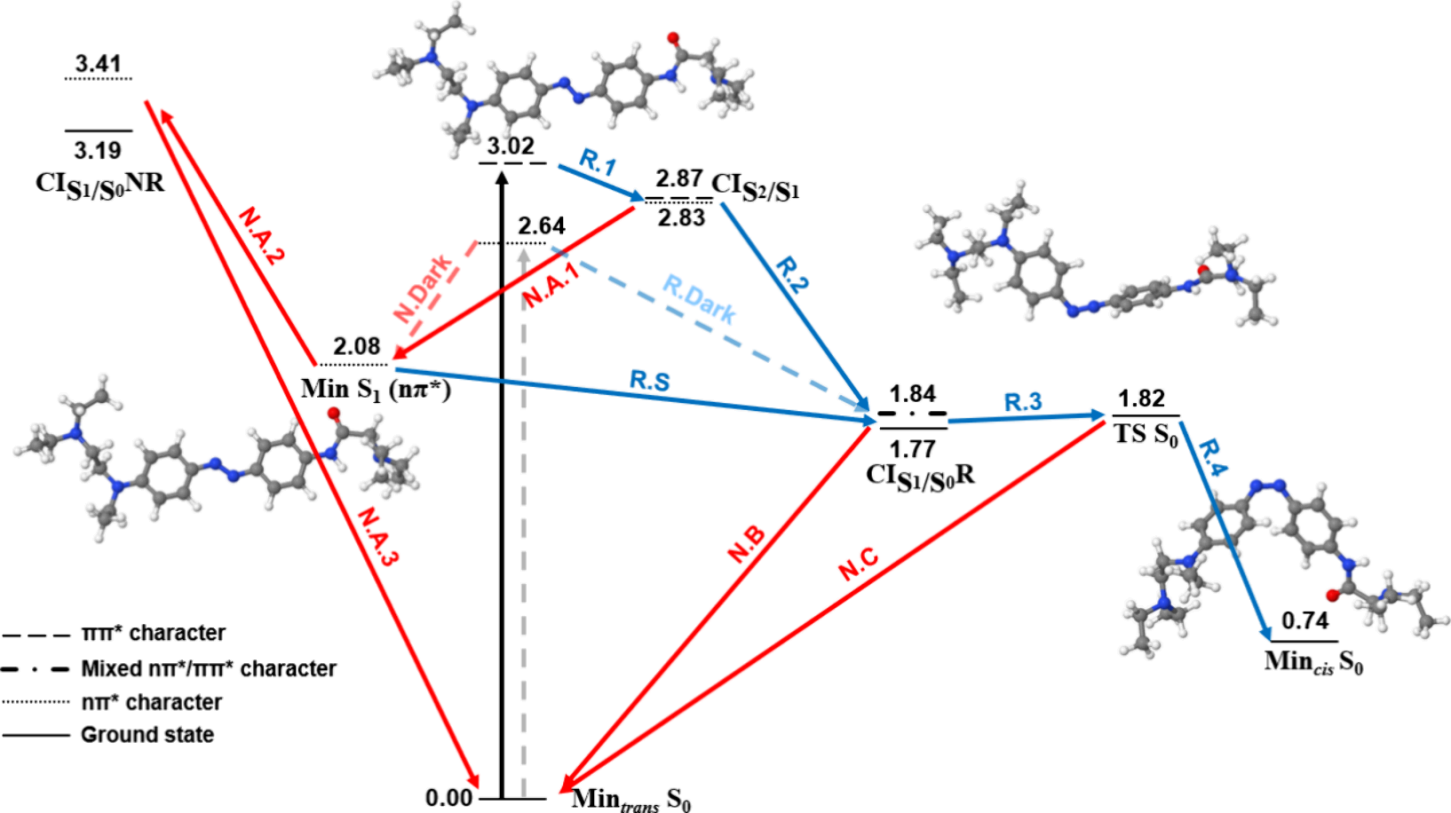
Relaxation pathways of *trans*-DAD in vacuum at B3LYP/cc-pVDZ. Blue and red arrows indicate reactive (R) and nonreactive pathways (N), respectively. Continuous and dashed arrows represent the bright ππ* and dark nπ* excitations, respectively, and their corresponding relaxation pathways. Energies in eV relative to Min_*trans*_*S*_0_.

**Figure 6:**
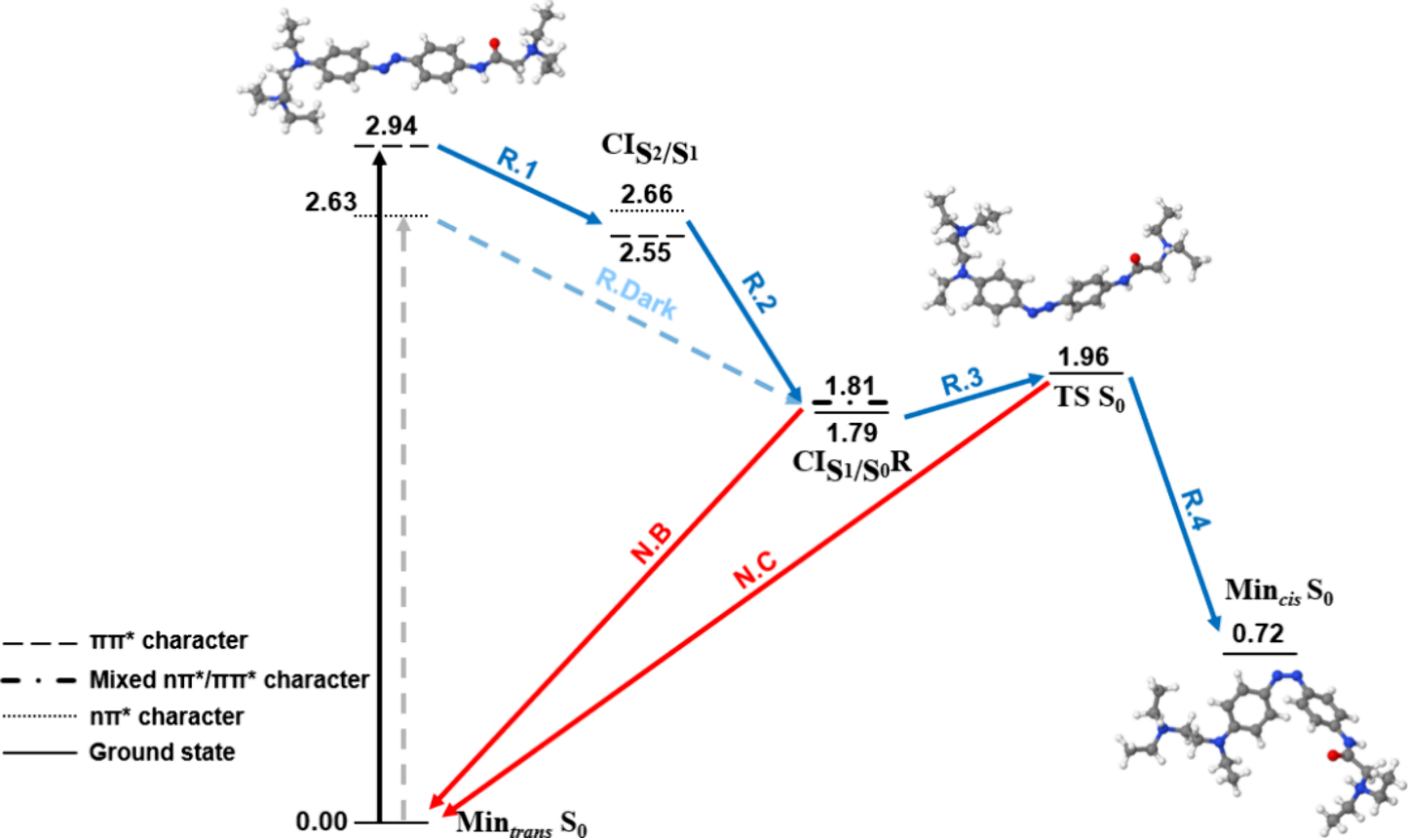
Relaxation pathways of *trans*-DADH_2_^+2^ in water at B3LYP/cc-pVDZ. Blue and red arrows indicate reactive (R) and nonreactive pathways (N), respectively. Continuous and dashed arrows represent the bright ππ* and dark nπ* excitations, respectively, and their corresponding relaxation pathways. Energies in eV relative to Min_*trans*_*S*_0_.

**Figure 7:**
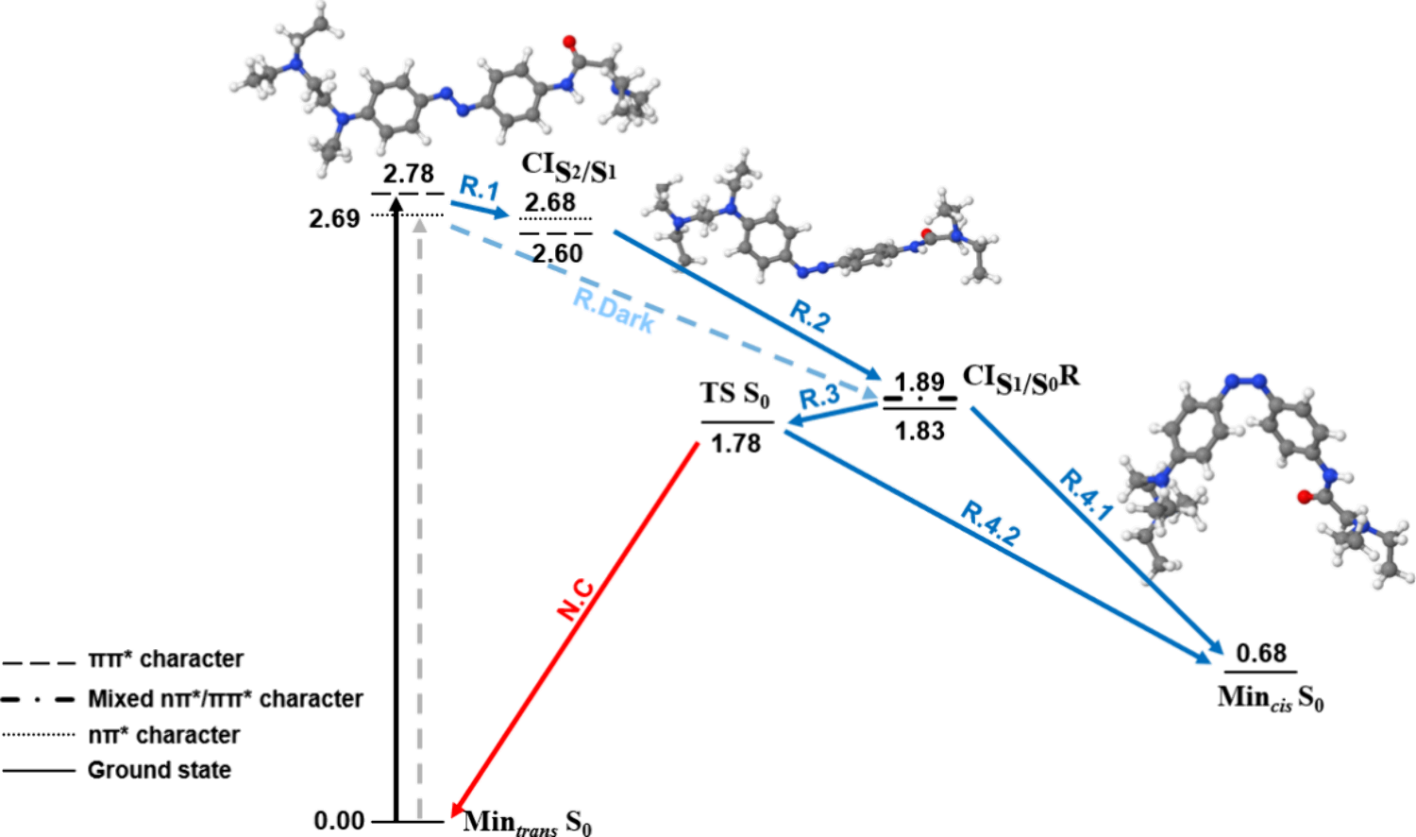
Relaxation pathways of *trans*-DAD in water at B3LYP/cc-pVDZ. Blue and red arrows indicate reactive (R) and nonreactive pathways (N), respectively. Continuous and dashed arrows represent the bright ππ* and dark nπ* excitations, respectively, and their corresponding relaxation pathways. Energies in eV relative to Min_*trans*_ S_0_.

The conformational space of the neutral and diprotonated *cis* and *trans* DAD was sampled with CREST^48^ in water. To see the effect of the conformation on the photophysics, we selected the 10 most representative CREST conformers out of all the resulting conformations for each system (see Extended Computational Details in SI), and we calculated their single point TDDFT absorption spectra in water, using the B3LYP,^49−52^ CAM-B3LYP^53^ and M06-2X^54^ functionals, and the cc-pVDZ^55^ basis set implemented in Gaussian16^56^ (see Figure S1 of the SI). The lowest energy conformers of each system were further optimized and their TDDFT absorption spectra were recalculated in water to do the benchmarking in the higher-level optimized structures (Figure 2A–D). Next, the absorption spectra were also computed considering an ensemble of geometries, i.e., ensemble spectra, of *cis* and *trans* DADH_2_^2+^, the predominant protonation state of DAD at pH = 7. To do that, TDDFT absorption spectra calculations in water were performed on 100 equidistant snapshots taken from the last 100 ns of a single 120 ns classical trajectory of the corresponding system in water. We performed these calculations at the B3LYP and M06-2X/cc-pVDZ levels to be consistent with the previous benchmarking (Figure 2E,F). Finally, we computed the *trans*-*cis* TDDFT photoisomerization pathways in vacuum (AB, DAD and DADH_2_^2+^), and in water (DAD and DADH_2_^2+^), at the M06-2X (see SI Figures S3–S7 and Tables S2–S6) and the B3LYP/cc-pVDZ (Figures 3–7, Tables 1–3, and 5,6) levels in Gaussian16. To see a more detailed explanation of the computational methods, please refer to the Extended Computational Details section in the SI.

**Table 1:** Geometrical Parameters of the Azo Moiety (C–N=N–C Dihedral, and C–N=N and N=N–C Angles in °) at Relevant Points of the Deactivation Pathway of *trans*-AB in Vacuum at B3LYP/cc-pVDZ

	CI_*S*1/*S*0_NR	Min *S*_1_ (nπ*)	Min*_trans_* *S*_0_	Min *S*_2_ (ππ*)	CI_*S*2/*S*1_	CI_*S*1/*S*0_R	TS *S*_0_	Min*_cis_* *S*_0_
C–N=N–C	–179.9	180.0	180.0	180.0	180.0	93.7	77.4	9.6
C–N=N	144.9	130.3	114.8	111.7	103.1	119.0	117.2	124.1
N=N–C	144.9	130.3	114.8	111.7	103.1	143.3	179.5	124.1

**Table 2:** Geometrical Parameters of the Azo Moiety (C–N=N–C Dihedral, and C–N=N and N=N–C Angles in °) at Relevant Points of the Deactivation Pathway of *trans*-DADH_2_^+2^ in Vacuum at B3LYP/cc-pVDZ

	CI_*S*1/*S*0_NR	Min *S*_1_ (nπ*)	Min*_trans_* *S*_0_	Min *S*_2_ (ππ*)	CI_*S*2/*S*1_	CI_*S*1/*S*0_R	TS *S*_0_	Min*_cis_* *S*_0_
C–N=N–C	176.4	176.1	179.0	179.8	–179.3	–111.7	56.7	–12.1
C–N=N	143.1	130.4	114.7	111.9	106.2	128.5	118.8	126.2
N=N–C	144.4	130.9	114.7	113.7	109.7	133.0	177.5	126.8

**Table 3:** Geometrical Parameters of the Azo Moiety (C–N=N–C Dihedral, and C–N=N and N=N–C Angles in °) at Relevant Points of the Deactivation Pathway of *trans*-DAD in Vacuum at B3LYP/cc-pVDZ

	CI_*S*1/*S*0_NR	Min *S*_1_ (nπ*)	Min*_trans_* *S*_0_	CI_*S*2/*S*1_	CI_*S*1/*S*0_R	TS *S*_0_	Min*_cis_* *S*_0_
C–N=N–C	176.4	–176.1	179.9	179.7	105.9	–102.2	11.6
C–N=N	143.1	130.0	115.3	110.4	128.2	117.8	125.2
N=N–C	144.1	130.3	114.8	113.5	126.2	178.8	124.6

**Table 4:** Energy Gap and Bond Distances in the Azo Moiety (Figure 1) of *trans*-DAD, DADH_2_^2+^ and AB at the Optimized B3LYP/cc-pVDZ Structures in Vacuum

	*S*_2_*–S*_1_ gap (eV)	C–N dist (Å)	*N*=N dist (Å)	N–C dist (Å)
DAD	0.38	1.406	1.264	1.413
DADH_2_^2+^	0.79	1.415	1.259	1.417
AB	1.22	1.420	1.258	1.420

**Table 5:** Geometrical Parameters of the Azo Moiety (C–N=N–C Dihedral, and C–N=N and N=N–C Angles in °) at Relevant Points of the Deactivation Pathway of *trans*-DADH_2_^+2^ in Water at B3LYP/cc-pVDZ

	Min*_trans_* *S*_0_	CI_*S*2/*S*1_	CI_*S*1/*S*0_R	TS *S*_0_	Min*_cis_* *S*_0_
C–N=N–C	–179.0	177.8	–104.7	116.0	–11.3
C–N=N	115.5	113.8	125.5	118.4	125.5
N=N–C	114.8	114.5	128.5	179.2	124.3

**Table 6:** Geometrical Parameters of the Azo Moiety (C–N=N–C Dihedral, and C–N=N and N=N–C Angles in °) at Relevant Points of the Deactivation Pathway of *trans*-DAD in Water at B3LYP/cc-pVDZ

	Min*_trans_* *S*_0_	CI_*S*2/*S*1_	TS *S*_0_	CI_*S*1/*S*0_R	Min*_cis_* *S*_0_
C–N=N–C	180.0	180.0	–155.8	81.7	10.0
C–N=N	115.8	115.8	118.6	121.2	126.0
N=N–C	114.9	114.9	179.5	119.6	125.9

## Results and Discussion

### Absorption Spectrum

The 10 most representative structures from the CREST conformational search show very similar vertical absorption spectra for each species and level of theory (Figure S1). This is because, as illustrated in Figures S9 and S11–S13, the orbitals involved in the electronic transitions are localized in the azobenzene moiety, whereas the conformational changes mainly occur in the substituents of the aromatic rings. As a result, the corresponding wave function barely changes, and the character and the energy of the excitations remain almost equal no matter the conformation. This indicates that the different conformers adopted by each system would probably follow very similar photoisomerization pathways and, therefore, any of them would be suitable to proceed with our study. In this case, we selected the lowest energy representative structure of each species to continue with our calculations.

Figure 2A–D shows the vertical absorption spectra of the corresponding optimized structures in water. Upon comparison with the experimental spectra, B3LYP provides the most accurate results. On the other hand, CAM-B3LYP and M06-2X show very similar spectra, that are blue-shifted with respect to the experiment. The observed blue shift can be a consequence of lacking the coupling with the vibrational degrees of freedom. Hence, to obtain more realistic absorption spectra, 100 equidistant snapshots were extracted from the last 100 ns of the MD simulations of *cis* and *trans*-DADH_2_^+2^, the most abundant protonation state at pH = 7, in explicit water. From these geometries, single point excitations were calculated in implicit water (IEFPCM) at the B3LYP and M06-2X/cc-pVDZ levels of theory. Calculations at CAM-B3LYP were not performed because we expect similar results to M06-2X due to the previous M06-2X/CAM-B3LYP resemblance. The resulting spectra are shown in Figure 2E,F. Once again, there is a much better match between the B3LYP and the experimental spectra and, in fact, the spectral shape significantly improves in *cis*- DADH_2_^+2^, whereas M06-2X is still far from the experiment. Taking this into account, we selected the B3LYP functional to discuss the DAD photoisomerization pathways in the following sections. However, although the *S*_2_ state has no charge transfer character (Figures S8–S13), we identified partial intramolecular charge transfer in the *S*_1_ state and, thus, B3LYP could potentially fail in the description of the excited states. For this reason, we also performed the same calculations at the M06-2X level to compare them with the B3LYP results (see SI).

### Photoisomerization Mechanism

In the following, the photoisomerization mechanisms of DAD and DADH_2_^+2^ are analyzed in detail. First, we compare the main differences between both species and AB, i.e., we discuss the functionalization of AB with amino groups, and we explore the protonation effect. Then, the effect of solvating the chromophore is discussed by comparing the photoisomerization pathways of DAD and DADH_2_^+2^ in vacuum and in implicit solvent. Figures 3–7 show the photoisomerization pathways for all the investigated species with the character of the participating electronic states, and Figures 1 and S8–S13 show the involved orbitals in the electronic excitations of the *cis* and *trans* conformations. As illustrated, the evolution of the structures (Tables 1–3, 5 and 6) and the character of the states along the photoisomerization (Figures 3–7) are equivalent in all the systems. Hence, we provide the critical point *xyz* geometries (supplementary geomsDADprotvacuumB3LYP.xyz file) and the detailed excited states results (Table S7, Figures S10 and S14–S18) for the DADH_2_^2+^ PES in vacuum as a model example. Moreover, the *trans* species are more stable than their corresponding *cis* species. Therefore, the *trans* conformation will be more abundant in the dark and the photoisomerization will start by its absorption of light. In the *trans* conformation, there is only one bright excited state close to the visible region, corresponding to the *S*_2_ ππ* electronic transition (Figures S8–S11). Hence, most probably, the *S*_2_ state will be populated when irradiated at short enough wavelengths.

### Photoisomerization: Functionalization and Protonation Effects

Figure 3 shows the relaxation pathways of *trans*-AB in vacuum. Upon bright *S*_2_ ππ* excitation, the ππ* state geometry relaxes to a minimum (R.1.1), which is close to a crossing point with the nπ* state (R.1.2). Both geometries present no significant changes with respect to the Franck–Condon one. Then, the system may jump to the nπ* surface and, next, it may evolve through a nonreactive pathway (red arrows N.A.1–3) or a reactive pathway leading to isomerization (blue arrows R.2–4). From the *S*_2_/*S*_1_ crossing point, the geometry optimization evolved through the nonreactive pathway, leading to a nπ* minimum in which the azo C–N=N angles increase (N.A.1). This is close to a crossing point with the ground state (N.A.2), where the molecule relaxes back to the *trans* isomer (N.A.3). The nonreactive pathway was also found when relaxing the nπ* from the Franck–Condon region (N.Dark). Hence, as the population of the nπ* minimum is possible upon both bright and dark excitations, their different photoisomerization yields can only be explained with dynamical studies. On the other hand, the reactive pathway could only be followed by optimizing a structure, that resembles the *S*_2_/*S*_1_ crossing point, in which the dihedral of its azo moiety was manually rotated. As a result, based on these energetic considerations, it qualitatively seems that the nonreactive pathway is more likely than the reactive one. Note that, to quantify it, excited state dynamic simulations would be required. Along the reactive pathway, the system relaxes to a crossing point with the ground state (R.2), which is close to the *trans*-*cis* transition state (TS) in the ground state (R.3). Hence, if it crosses to the ground state and it reaches the TS, it may photoisomerize to the *cis*-AB (R.4). Alternatively, it may also go back to the *trans*-AB (N.B–C). When relaxing in the nπ* surface, the C–N=N–C dihedral rotates and reaches 93.7° in the crossing point with the ground state. The dihedral is then 77.4° in the TS, which continues evolving until 9.6° in the *cis* minimum (Table 1).

DADH_2_^2+^ in vacuum follows a very similar landscape (Figure 4 and Table 2). We observe the main differences with AB in the Franck–Condon region. In here, the *S*_1_ nπ* state energy remains almost equal (∼2.5 eV), whereas the *S*_2_ ππ* significantly lowers its energy from 3.76 eV in *trans*-AB to 3.29 eV in *trans*-DADH_2_^+2^. This leads to a reduction in the *S*_2_–*S*_1_ energy gap and, thus, there is less excess energy in the DADH_2_^+2^ relaxation, and the population of alternative pathways in the photoisomerization might be less likely. Moreover, the required excitation wavelength is less harmful, since it is less energetic, and it is closer to the visible region. This energy difference seems to also change the PES profile beyond the Franck–Condon region. Compared to AB, the ππ* minimum in DADH_2_^+2^ is closer to its crossing with the nπ* state (R.1.2). As a result, DADH_2_^+2^ will probably not stay long at the minimum before reaching the crossing point. On the other hand, we observe a deeper minimum with a larger energy difference between the ππ* minimum and the crossing point with nπ* in AB. Hence, the excited AB molecule may stay in the minimum long enough to deactivate through different pathways that could become accessible due to its vibrational energy excess, as suggested by previous experimental studies.^18^ All these reasons indicate a potentially higher photoisomerization quantum yield for DADH_2_^+2^.

By comparing Figures 4 (DADH_2_^+2^) and 5 (DAD), and Tables 2 and 3, the effect of protonation on the photophysics of the chromophore is analyzed. Upon protonation, the nπ* energy decreases (from 2.64 to 2.50) and the ππ* energy increases (from 3.02 to 3.29 eV), leading to a larger *S*_2_–*S*_1_ gap in the Franck–Condon region of DADH_2_^2+^. Considering that one of the main goals of the derivation of AB is red shifting the ππ* energy and decreasing the gap, it seems that the effect of the functional amino groups is less noticeable in DADH_2_^2+^ than in DAD. In this sense, the resulting AB-DADH_2_^2+^-DAD *S*_2_–*S*_1_ energy gap order can be related to the change in the geometry of the azo moiety (C–N=N–C), as shown in Table 4. According to these results, larger N=N bonds and shorter C–N bonds may favor the desired ππ* red shifting and the *S*_2_–*S*_1_ energy gap decrease. Therefore, the protonation state of DAD could affect the excitation energies, and the photoisomerization quantum yield, due to the structural change that it produces.

Moreover, no ππ* minimum appears in the DAD surface in vacuum. This change in the PES is probably induced by the initial closeness between the *S*_2_ and *S*_1_ states in the Franck–Condon region. Hence, the excited molecule relaxes directly to the ππ*/nπ* crossing point (R.1). Then, it can follow the nonreactive (N.A.1–3) or the reactive pathways (R.2–4). According to the SHARC calculation from the nπ* minimum in the nonreactive pathway (N.A.1), the molecule can return to the reactive pathway by reaching a crossing point with the ground state (R.S) from which the *trans*-*cis* TS is accessible (R.3). In this case, evolution through the R.S path might be more likely due to the high energetic barrier that is required to reach the nonreactive crossing with the ground state (N.A.2). Finally, this possibility is further explored in the SI by a NEB calculation that estimates an energetic barrier smaller than 0.2 eV in the geometry interpolation along the R.S path (Figure S19).

### Photoisomerization: Solvation Effect

Finally, we now discuss the influence of the solvent on the photoisomerization mechanism of DADH_2_^+2^ and DAD. Upon implicit solvation (Figures 6, 7, Tables 5 and 6), there is a significant reduction in the *S*_2_–*S*_1_ energy gap in the Franck–Condon region. This time, not only the ππ* energy decreases (from 3.29 to 2.94 eV in DADH_2_^2+^ and from 3.02 eV to 2.78 in DAD), but also the nπ* energy increases (from 2.50 to 2.63 eV in DADH_2_^2+^ and from 2.64 eV to 2.69 in DAD). As a result, the gap between both electronic states decreases in water. Once again, this is convenient to avoid the electronic relaxation through pathways different from the *trans*-*cis* photoisomerization after excitation to the bright ππ* state. Furthermore, the ππ* minimum disappears in water and the geometry relaxes directly to the crossing point with nπ* (R.1). Moreover, when crossing to the nπ* surface, the optimizations led to the *S*_1_ crossing with the ground state (R.2), instead of reaching a nonreactive nπ* minimum as observed in vacuum (N.A.1 Figures 3–5). Therefore, the molecules seem to evolve more favorably through the reactive pathway (R.2–4). Compared to DADH_2_^2+^, the nonprotonation of DAD, combined with the solvation effect, transforms the DAD Franck–Condon region into an almost ππ*/nπ* crossing point. In addition, the geometry of DAD in the crossing point with the ground state relaxes directly to *cis*-DAD (R.4.1), whereas in DADH_2_^2+^ it led to the *trans* isomer (N.B). Based on all these energetic considerations, it qualitatively seems that DAD in water will probably present the best photoisomerization yield among the considered systems. However, as mentioned earlier, dynamic calculations would be required to quantify it.

## Conclusions

In this work, we presented the first computational study of the absorption and photoisomerization mechanism of the DAD photoswitch, to our knowledge. Previous experimental work^1,46,47^ already proved the potential of DAD to control biological functions with light. Here, we showed that single point calculations for the most representative *cis*/*trans* DAD/DADH_2_^2+^ conformers, along with the computation of the ensemble absorption spectra, indicated that B3LYP/cc-pVDZ is an appropriate level of theory to study the DAD photoisomerization, and that any of the found conformers is a reasonable starting point for this purpose. In general terms, AB, DAD and DADH_2_^2+^ populate the bright *S*_2_ ππ* excited state when irradiated at short enough wavelengths. Then, they relax to the nπ* surface in which the azo dihedral evolves until reaching a crossing point with the ground state, that is very close to the *trans*-*cis* TS. From here, the system can relax to the *cis* isomer. The comparison of AB, DAD and DADH_2_^2+^ suggests that the key points in the photoisomerization are the *S*_2_–*S*_1_ energy gap, the presence of a minimum in the ππ* surface prior to its crossing point with the nπ* state, and the existence of a nonreactive deactivation pathway. This depends on the derivation of AB, the protonation effect and on solvation. Derivation, nonprotonation and solvation favor the reduction of the *S*_2_–*S*_1_ energy gap and induce the disappearance of the ππ* minimum. In addition, although dynamic excited states simulations would be required to quantify it, the nonreactive pathway seems to be more favorable in nonsolvated systems and not predominant in solvated systems. As a result, solvated DAD would probably yield the most efficient photoisomerization among the considered systems, given the small *S*_2_–*S*_1_ energy gap, and the absence of the ππ* minimum and nonreactive pathway. We expect DADH_2_^2+^, the predominant species at biological pH, to be slightly less efficient than DAD, but significantly better than AB. From these results, it is clear that the environment plays a crucial role in the photoisomerization of DAD. Hence, future works could focus on its study in a biological environment, such as an ion channel. Moreover, we also noted the relevance of the differences in bond lengths of the azo moiety among the species in the Franck–Condon region, as larger N=N bonds and shorter C–N bonds, seem to red shift the ππ* state and lead to a *S*_2_–*S*_1_ energy decrease. In this sense, it would also be interesting to deepen more into this and to extend the study to other AB derivatives in future investigations.

## Supporting Information Available

geomsDADprotvacuumB3LYP.xyz: Critical geometries of the DADH_2_^2+^ in vacuum relaxation pathways (XYZ)
Extended computational details, additional figures of the absorption spectra of the CREST representative structures, ensemble absorption spectra QM/MM vs QM/PCM and photoisomerization pathways at the M06-2X/cc-pVDZ level, detailed excited state results of DADH_2_^2+^ in vacuum, and geometry interpolation between two points in the DAD in vacuum excited states relaxation (PDF)

## Supplementary Material

jp4c05179_si_001.xyz
