# Supplementary material for: Computational Characterization of the DAD Photoisomerization: Functionalization, Protonation, and Solvation Effects
Source: J Phys Chem B. 2024 Nov 16;128(47):11587–96. doi: 10.1021/acs.jpcb.4c05179 (PMC11613546; doi:10.1021/acs.jpcb.4c05179)
Supplement: Supplementary file 2 — jp4c05179_si_002.pdf [file jp4c05179_si_002.pdf]

Supporting Information for Computational characterization of the DAD photoisomerization: Functionalization, protonation and solvation effects.

Lucía López-Pacios[a], *Juan J. Nogueira*[a,b]*, *and Lara Martínez-Fernández*[c]*

[a]Departamento de Química, Facultad de Ciencias, Universidad Autónoma de Madrid, Campus de Excelencia UAM-CSIC, Cantoblanco, 28049 Madrid, Spain.

[b]Institute for Advanced Research in Chemistry (IAdChem), Universidad Autónoma de Madrid, Campus de Excelencia UAM-CSIC, Cantoblanco, 28049 Madrid, Spain.

[c]Departamento de Química Física de Materiales, Instituto de Química Física Blas Cabrera, CSIC, 28006 Madrid, Spain.

S1) Extended Computational Details

Benchmarking and Conformational Sampling

DAD and $DADH_2^{2+}$ are large molecules containing 73 and 75 atoms respectively. Due to the large number of possible degrees of freedom, the absorption spectrum will depend on the conformations that it can adopt. To see this effect, the conformational space of *cis* and *trans* DAD and $DADH_2^{2+}$ was sampled with CREST[1] in water, using the GBSA implicit solvation model[2], the GFN2-xTB[3]//GFN-FF[4] composite method and constrains in the azo dihedral to keep the *cis* or *trans* conformation during the conformational search. The CREST calculation rendered 1316, 2605, 778

and 2320 conformers for *cis*-DAD, *trans*-DAD, *cis*-$DADH_2^{2+}$ and *trans*-$DADH_2^{2+}$, respectively. To simplify the analysis and reduce the computational cost, we clustered all the resulting conformers for each system in the 10 most representative structures, all belonging to the original configuration space, using the k-means algorithm[5] implemented in cpptraj[6]. Then their absorption spectra were obtained:

1) To select the most appropriate representative structure: Single point time-dependent density functional theory (TDDFT) calculations of the 10 representative structures (optimized at the GFN2-xTB level during the CREST sampling) in water (non-equilibrium IEFPCM[7–9]), using the B3LYP[10–13], CAM-B3LYP[14] and M06-2X[15] functionals and the cc-pVDZ[16] basis set implemented in Gaussian16[17] (Figure S1). We selected these functionals because of their good performance in the excited states calculations in previous studies[18]. Results in Table S1 and Figure S1 show small RMSD and absorption spectrum differences among the representative structures, and a small number of conformations in cluster 9, indicating that the clustering is well converged by requesting 10 structures. The resemblance of the absorption spectra in Figure S1 indicate that any of these structures would be valid to proceed with our studies. For this reason, we selected the lowest energy representative structures for each system as we assumed they would be closer to the absolute minimum in the ground state.

2) To select the most appropriate TDDFT functional: We decided to further optimize the selected DAD and $DADH_2^{2+}$ GFN2-xTB structures at the higher levels of the theory B3LYP, CAM-B3LYP and M06-2X/cc-pVDZ in implicit water. Then, single point TDDFT calculations were performed in water at the B3LYP, CAM-B3LYP and M06-2X/cc-pVDZ levels of theory to do the benchmarking in the higher-level optimized structures (see results in Figure 2A-D in the main manuscript).

Table S1. RMSD with respect to the representative structure of cluster 0 and distribution (number of configurations per cluster, n° conf.) of the conformers in the k-means clustering of *cis* and *trans* DAD and $DADH_2^{2+}$.

	cis-DAD		*trans*-DAD		*cis*-$DADH_2^{2+}$		*trans*-$DADH_2^{2+}$	
Cluster	n° conf.	RMSD	n° conf.	RMSD	n° conf.	RMSD	n° conf.	RMSD
0	681	0	483	0	396	0	434	0
1	210	2.06	444	1.98	87	1.50	412	2.41
2	99	2.67	371	1.94	76	1.61	393	2.05
3	85	2.39	331	1.46	61	2.97	258	2.33
4	68	3.04	288	2.21	56	1.61	230	2.07
5	63	2.62	263	1.64	25	3.56	213	1.88
6	42	3.15	213	2.21	24	3.12	200	1.61
7	32	3.05	193	1.69	23	3.28	153	1.60
8	18	2.88	14	2.17	21	3.24	26	1.89
9	18	2.28	5	1.55	9	2.69	1	1.57

Next, we calculated the absorption spectra considering an ensemble of geometries, i.e. ensemble spectra, of *cis* and *trans* $DADH_2^{2+}$, the predominant protonation state of DAD at pH=7. To do that, TDDFT absorption spectra calculations in water were performed on 100 equidistant snapshots taken from the last 100 ns of a single 120 ns classical trajectory of the corresponding system in water. In particular, the next protocol was followed. The predominant protonation state of DAD at pH=7, which was $DADH_2^{2+}$, was calculated with obabel[19]. Next, the restrained electrostatic potential (RESP) charges were calculated in vacuum at the Hartree-Fock[20]/6-31G*[21–23] level of theory for *cis* and *trans* $DADH_2^{2+}$, at their previously optimized B3LYP structures. Then, the optimized

B3LYP/cc-pVDZ $DADH_2^{2+}$ was surrounded by water in a periodic truncated octahedral box, using the *tleap* module included in AmberTools20[24]. The water molecules were described by the TIP3P force field[25] and were present within 12 Å from any $DADH_2^{2+}$ atom. Moreover, 2 Cl^- ions were added to neutralize the system, that were also described by TIP3P. Finally, the DAD parameters were described by the GAFF2 force field[26], except for the charges, that were the previously calculated RESP charges, and they were generated with Antechamber and parmchk2 (Amber Tools20). Next, MD simulations were performed with Amber 20. First, the system was minimized for 2500 steps using the steepest descent algorithm, followed by 2500 steps using the conjugate gradient method. Then, the system, whose initial velocity was randomly generated according to a Boltzmann distribution, was progressively heated from 0 K to 303.15 K in the NVT ensemble with a 2 fs time-step, and using the Langevin thermostat[27] with 1.0 ps^{-1} collision frequency for 500 ps. Finally, a 2 fs time-step production, starting from the last heating coordinates and velocities, was performed in the NPT ensemble for 120 ns. Once again, the temperature was controlled with the Langevin thermostat (1.0 ps^{-1}) at 303.15 K. Likewise, the pressure was controlled with the Berendsen barostat[28] at 1.0 bar. In both, the heating and the production, the bonds involving hydrogen were constrained using SHAKE[29], and the azo dihedrals were constrained according to reference[30] to keep DAD in the corresponding *cis* or *trans* conformation. The electrostatic interactions were computed by the Particle-Mesh Ewald summation[31] with the default grid spacing of 0.5 Å, and a cutoff of 8 Å was considered to calculate the nonbonded interactions. Next, the script main_qminputs.py from MoBioTools[32] was used to extract 100 equidistant snapshots from the last 100 ns of the production. Then, TDDFT/PCM calculations (water, non-equilibrium IEFPCM) at the B3LYP and M06-2X/cc-pVDZ levels of theory in Gaussian16 were performed in these frames. Here, we removed the explicit water molecules, and we included the implicit environment to be consistent with the single point optimized geometry spectra and the

photoisomerization PES calculations. In these one-geometry calculations, we considered that an implicit environment was more appropriate since it avoids the results dependence on the explicit water configuration while it also considers mutual solute-solvent polarization. Moreover, to check the effectiveness of the TDDFT/PCM model on the calculation of the ensemble spectra compared to TDDFT/MM, we also simulated the QM/MM ensemble absorption spectrum of *trans*-$DADH_2^{2+}$ in explicit water, in which the QM region consisted of *trans*-$DADH_2^{2+}$ at the M06-2X/cc-pVDZ level of theory. According to Figure S2, TDDFT/PCM shows slightly better results than TDDFT/MM, indicating that TDDFT/PCM is effective to the current system. Nevertheless, regarding the propagation of the MD trajectory, we decided to include the explicit water molecules to consider the effect that the different water configurations have on the $DADH_2^{2+}$ dynamics. Finally, to plot both one-geometry and ensemble spectra, a convolution of gaussians with a width of 0.3 eV was performed.

Photoisomerization

The *trans-cis* photoisomerization pathways in gas phase (AB, DAD and $DADH_2^{2+}$), and in implicit IEFPCM water (DAD and $DADH_2^{2+}$), were calculated with TDDFT at the M06-2X (Figures S3-7) and the B3LYP/cc-pVDZ (Figures 2-7) levels in Gaussian16. Based on the results of the benchmarking (Absorption Spectrum section in the Results and Discussion of the main manuscript), we focused the main discussion on the B3LYP results and their differences with M06-2X are indicated here in the SI. From the previously optimized DAD structures, geometry optimizations of the bright (S_2) and dark (S_1) excited states were performed until a minimum or a crossing with the S_1 (or S_0) was reached. Once in the S_0, the *trans-cis* transition state (TS) structure was optimized. Then, an Intrinsic Reaction Coordinate (IRC) calculation was performed to connect the TS with the *cis* and *trans* conformations. In the cases where the minimization of the excited

states led to a minimum, SHARC[33,34], interfaced between the energies and gradients of Gaussian16 and the optimizer of ORCA 5.0[35] at the corresponding B3LYP or M06-2X/cc-pVDZ levels of theory, was used to optimize the S_2/S_1 or S_1/S_0 intersections. Note that, in the present study, we want to obtain an estimate of the degeneracy regions to compare the trends in a qualitative way. Hence, although it is well known that TDDFT might fail in accurately describing the S_1/S_0 intersections, it is sufficient for our purposes considering the resemblance between the calculated TDDFT PES of azobenzene (Figures 3 and S3) and previous CASPT2 studies[36–38]. Equilibrium solvation was used in the PCM excited states optimizations. Furthermore, Nudged Elastic Band (NEB) calculations in ORCA 5.0, requesting 4 intermediate structures, were performed to connect certain points in the S_1 surfaces (see a more detailed explanation below Figure S19). Finally, Jmol[39] was used to generate the computational figures in this work.

A) *cis*-DAD B3LYP B) *trans*-DAD B3LYP

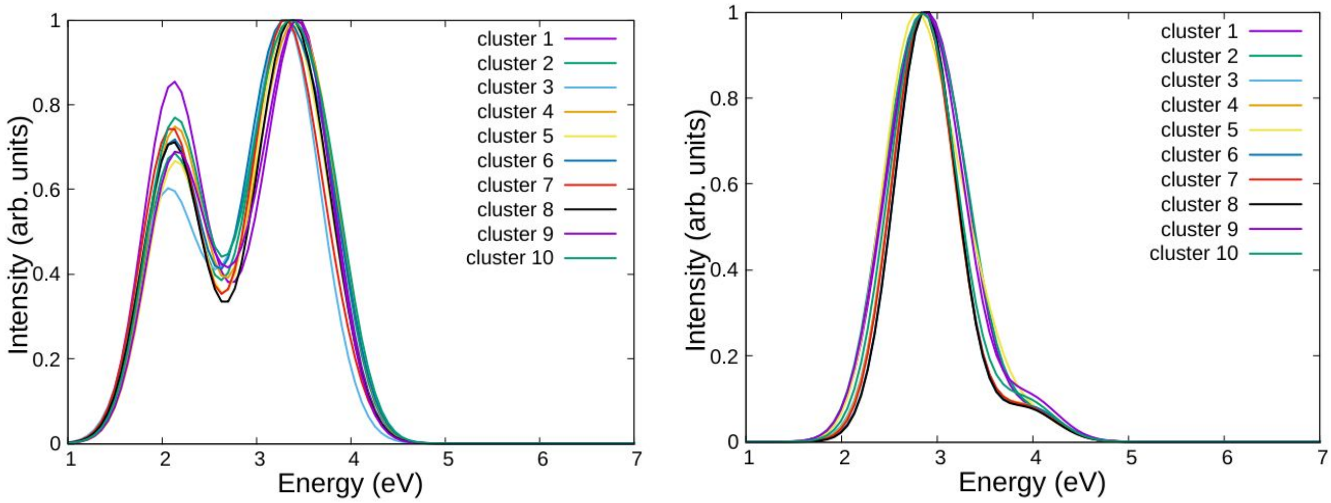

C) *cis*-DAD CAM-B3LYP D) *trans*-DAD CAM-B3LYP

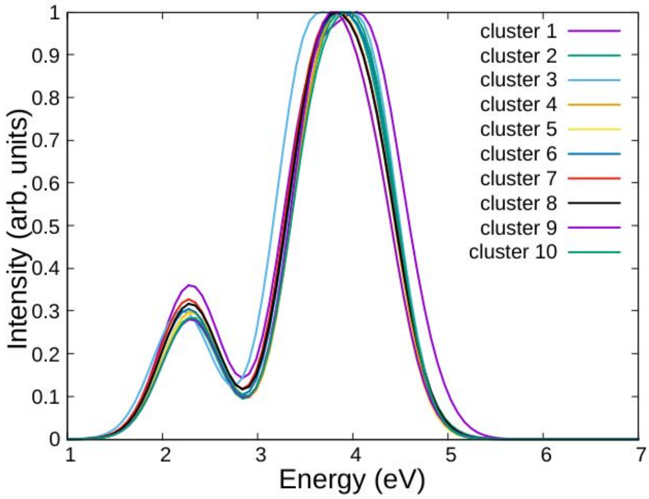

E) *cis*-DAD M06-2X

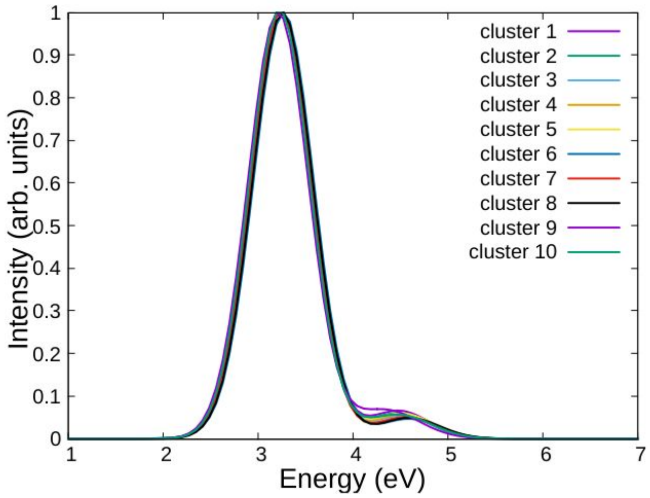

F) *trans*-DAD M06-2X

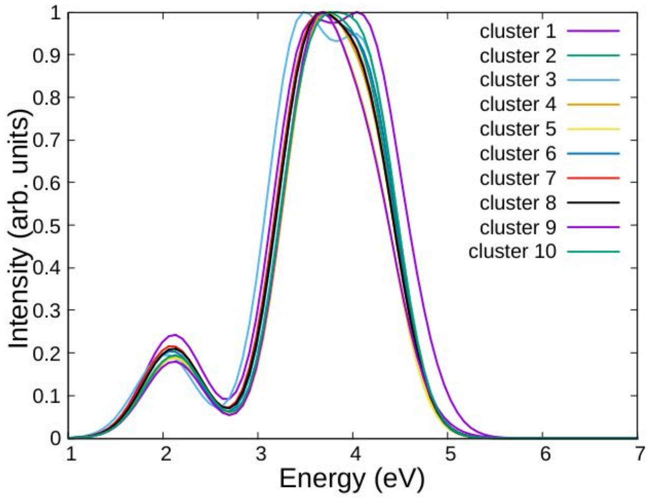

G) *cis*-$DADH_2^{2+}$ B3LYP

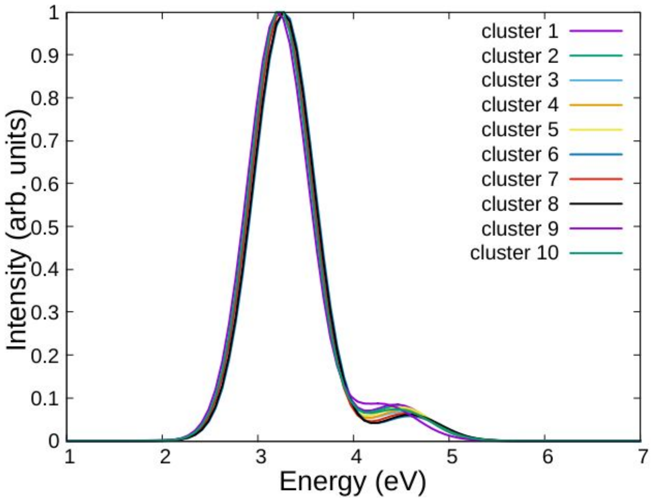

H) *trans*-$DADH_2^{2+}$ B3LYP

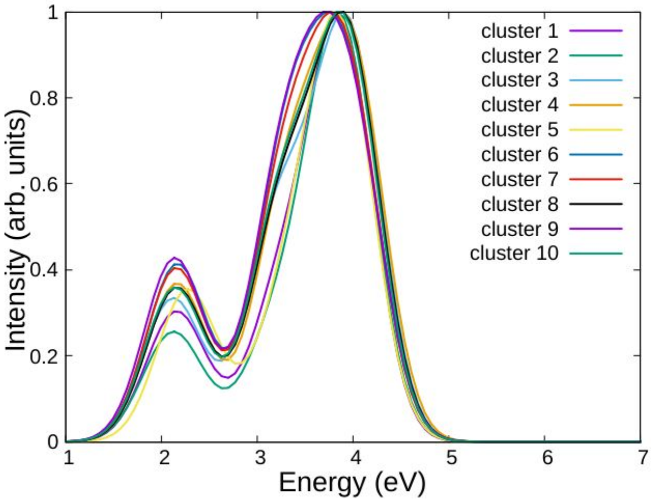

I) *cis*-$DADH_2^{2+}$ CAM-B3LYP

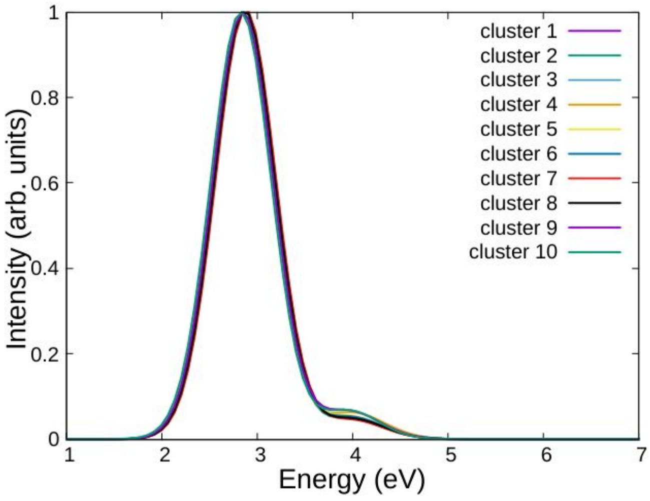

J) *trans*-$DADH_2^{2+}$ CAM-B3LYP

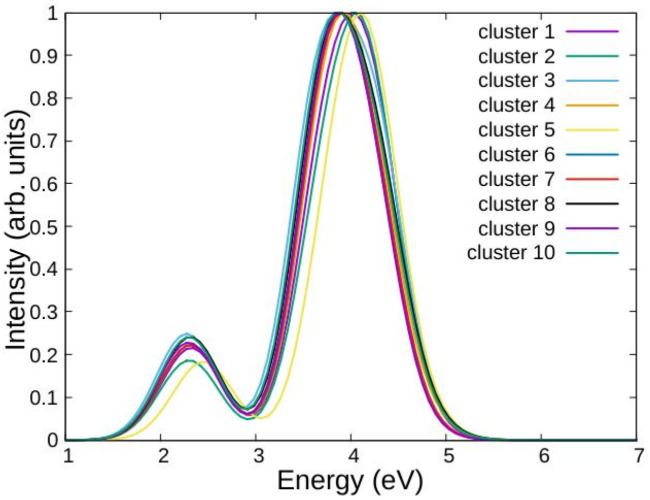

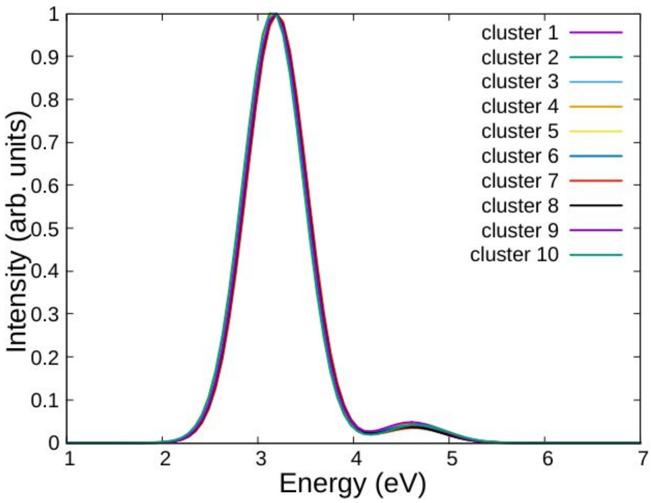

K) *cis*-$DADH_2^{2+}$ M06-2X

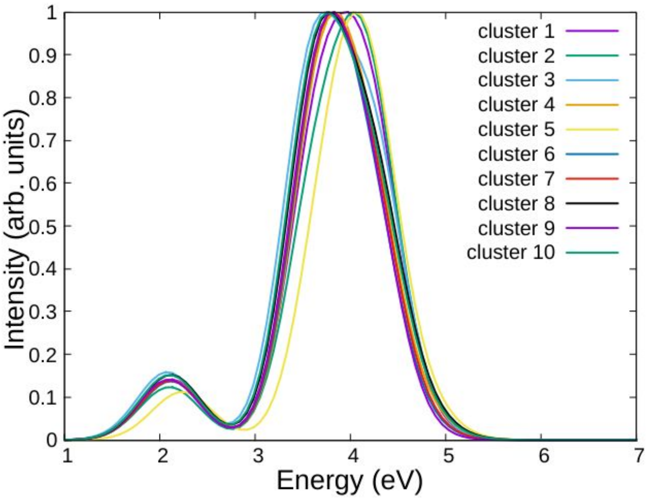

L) *trans*-$DADH_2^{2+}$ M06-2X

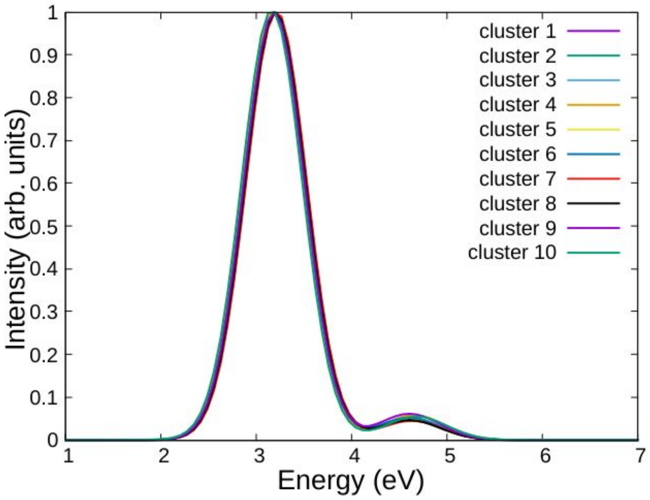

Figure S1. Vertical absorption spectra of the 10 most representative structures from the CREST conformational search in *cis* and *trans*, DAD and $DADH_2^{2+}$ in water, at the B3LYP, CAM-B3LYP and M06-2X/cc-pVDZ levels of theory.

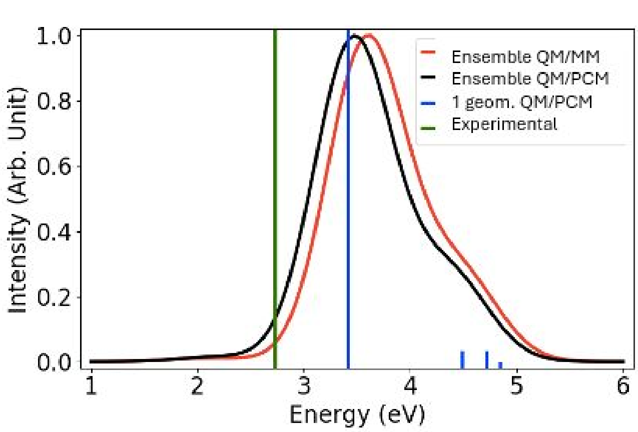

Figure S2. Ensemble absorption spectra of *trans*-$DADH_2^{2+}$ in water using the QM/MM and QM/PCM models (QM region at the M06-2X/cc-pVDZ level of theory). The one geometry spectrum and the experimental absorption maxima are indicated with lines.

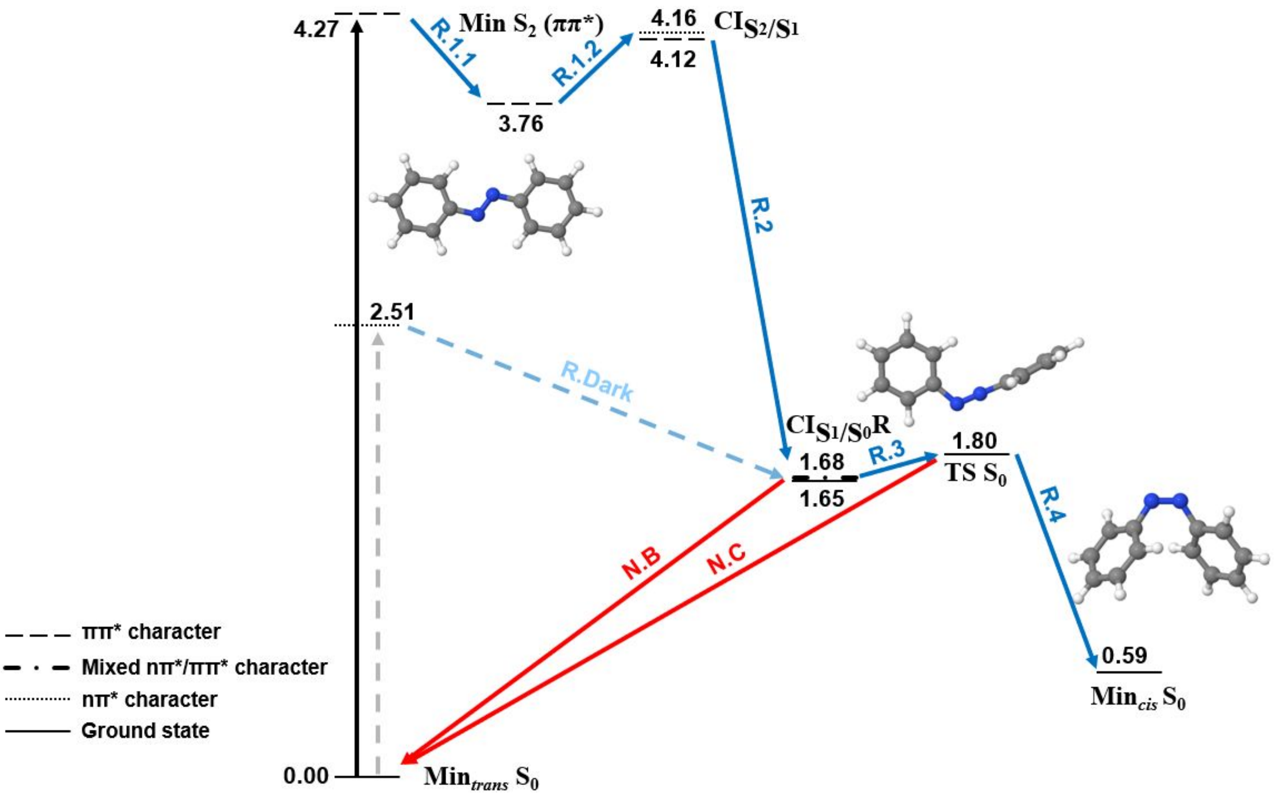

Figure S3. Relaxation pathways of *trans*-azobenzene in vacuum at M06-2X/cc-pVDZ. Blue and red arrows indicate reactive (R) and non-reactive pathways (N), respectively. Continuous and dashed arrows represent the bright ππ* and dark nπ* excitations, respectively, and their corresponding relaxation pathways. Energies in eV relative to Min_{trans} S_0.

Table S2. Geometrical parameters of the azo moiety (C-N=N-C dihedral, and C-N=N and N=N-C angles in °) at relevant points of the deactivation pathway of *trans*-azobenzene in vacuum at M06-2X/cc-pVDZ.

	Min_{trans} S_0	Min S_2 (ππ*)	$CI_{S2/S1}$	$CI_{S1/S0}$	TS S_0	Min_{cis} S_0

**C-N=N-C**	180.0	180.0	180.0	-151.4	88.0	7.2
**C-N=N**	114.4	111.7	102.2	136.2	116.4	122.6
**N=N-C**	114.4	111.7	102.2	136.2	180.0	122.6

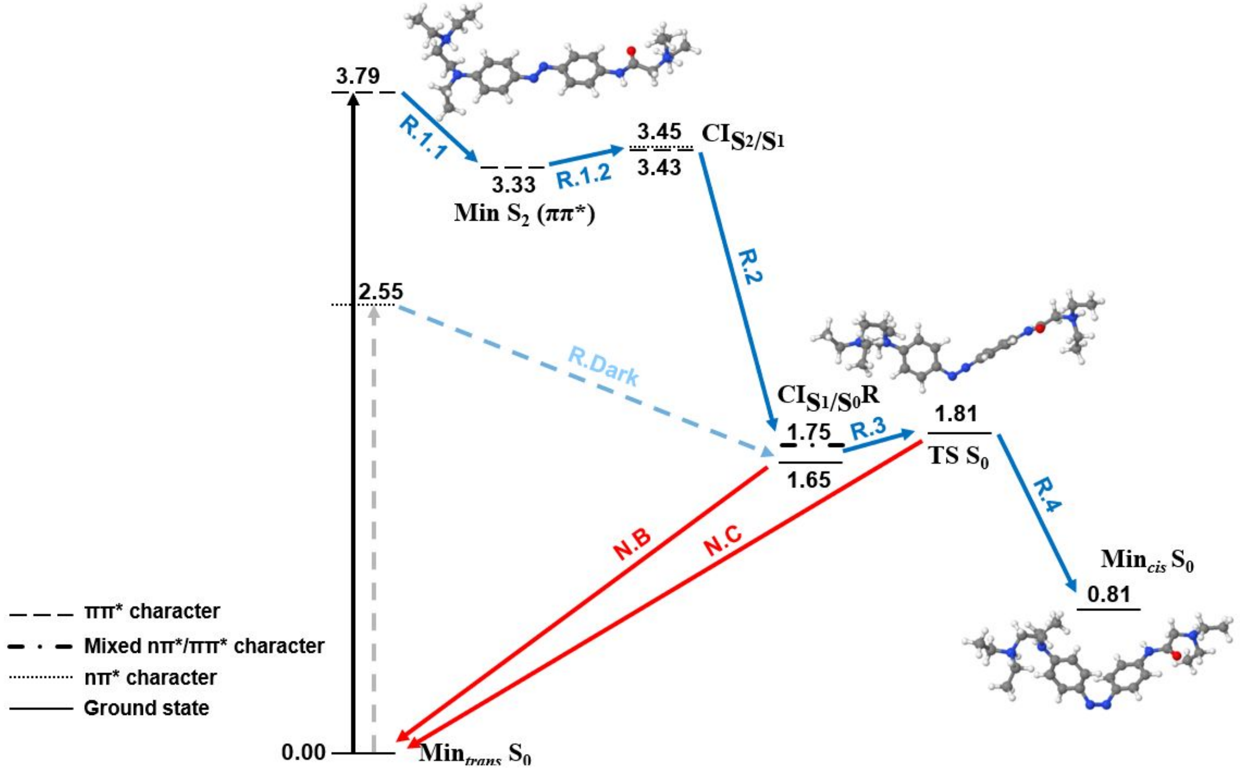

Figure S4. Relaxation pathways of *trans*-$DADH_2^{2+}$ in vacuum at M06-2X/cc-pVDZ. Blue and red arrows indicate reactive (R) and non-reactive pathways (N), respectively. Continuous and dashed arrows represent the bright ππ* and dark nπ* excitations, respectively, and their corresponding relaxation pathways. Energies in eV relative to Min_{trans} S_0.

Table S3. Geometrical parameters of the azo moiety (C-N=N-C dihedral, and C-N=N and N=N-C angles in °) at relevant points of the deactivation pathway of *trans*-$DADH_2^{+2}$ in vacuum at M06-2X/cc-pVDZ.

	**Min_{trans} S_0**	**Min S_2 (ππ*)**	**$CI_{S2/S1}$**	**$CI_{S1/S0}$**	**TS S_0**	**Min_{cis} S_0**

**C-N=N-C**	-179.2	-178.6	-179.1	-139.1	-54.1	-9.7
**C-N=N**	114.6	112.0	106.9	135.8	117.0	124.1
**N=N-C**	114.0	112.7	108.0	134.4	178.1	124.1

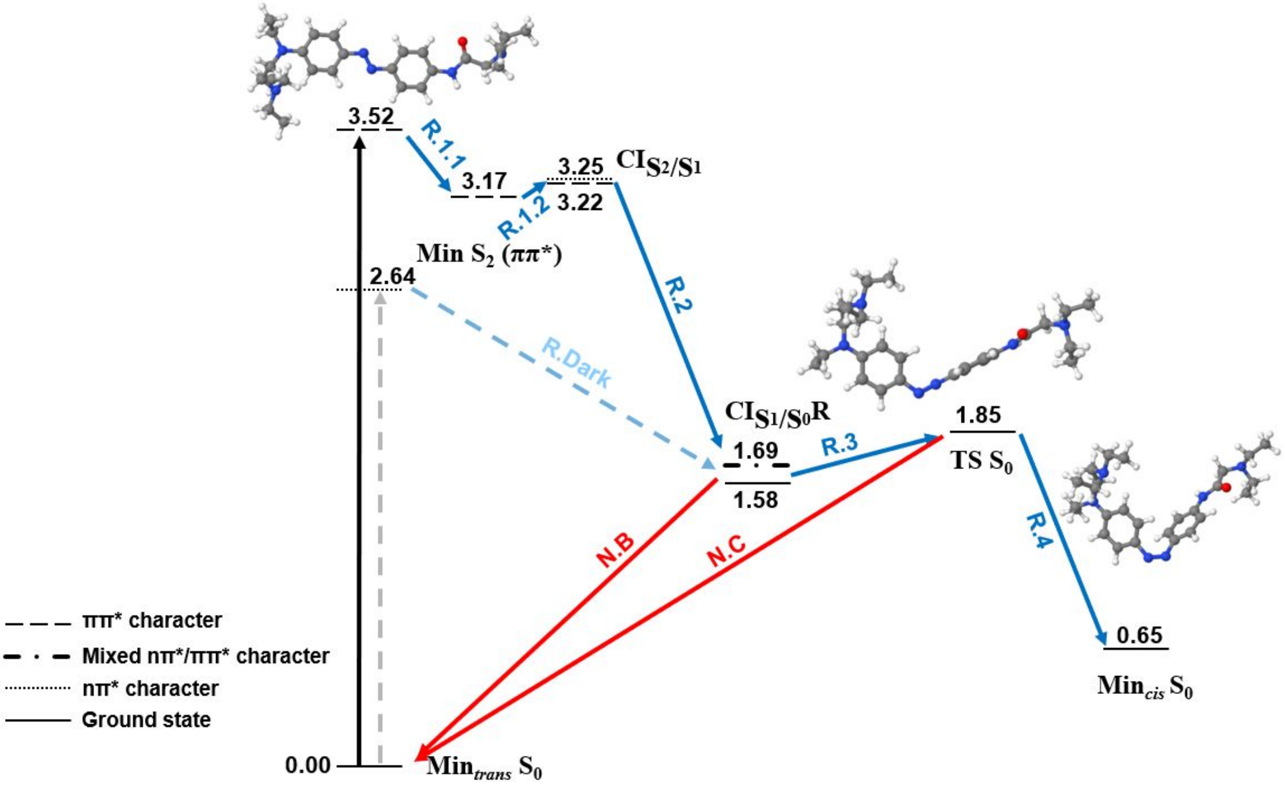

Figure S5. Relaxation pathways of *trans*-DAD in vacuum at M06-2X/cc-pVDZ. Blue and red arrows indicate reactive (R) and non-reactive pathways (N), respectively. Continuous and dashed arrows represent the bright ππ* and dark nπ* excitations, respectively, and their corresponding relaxation pathways. Energies in eV relative to Min_{trans} S_0.

Table S4. Geometrical parameters of the azo moiety (C-N=N-C dihedral, and C-N=N and N=N-C angles in °) at relevant points of the deactivation pathway of *trans*-DAD in vacuum at M06-2X/cc-pVDZ.

	**Min_{trans} S_0**	**Min S_2 (ππ*)**	**$CI_{S2/S1}$**	**$CI_{S1/S0}$**	**TS S_0**	**Min_{cis} S_0**

**C-N=N-C**	179.8	179.8	-179.6	-122.0	140.5	-8.9
**C-N=N**	114.9	111.8	108.2	129.6	116.5	123.4
**N=N-C**	114.2	113.1	109.6	127.8	179.0	122.9

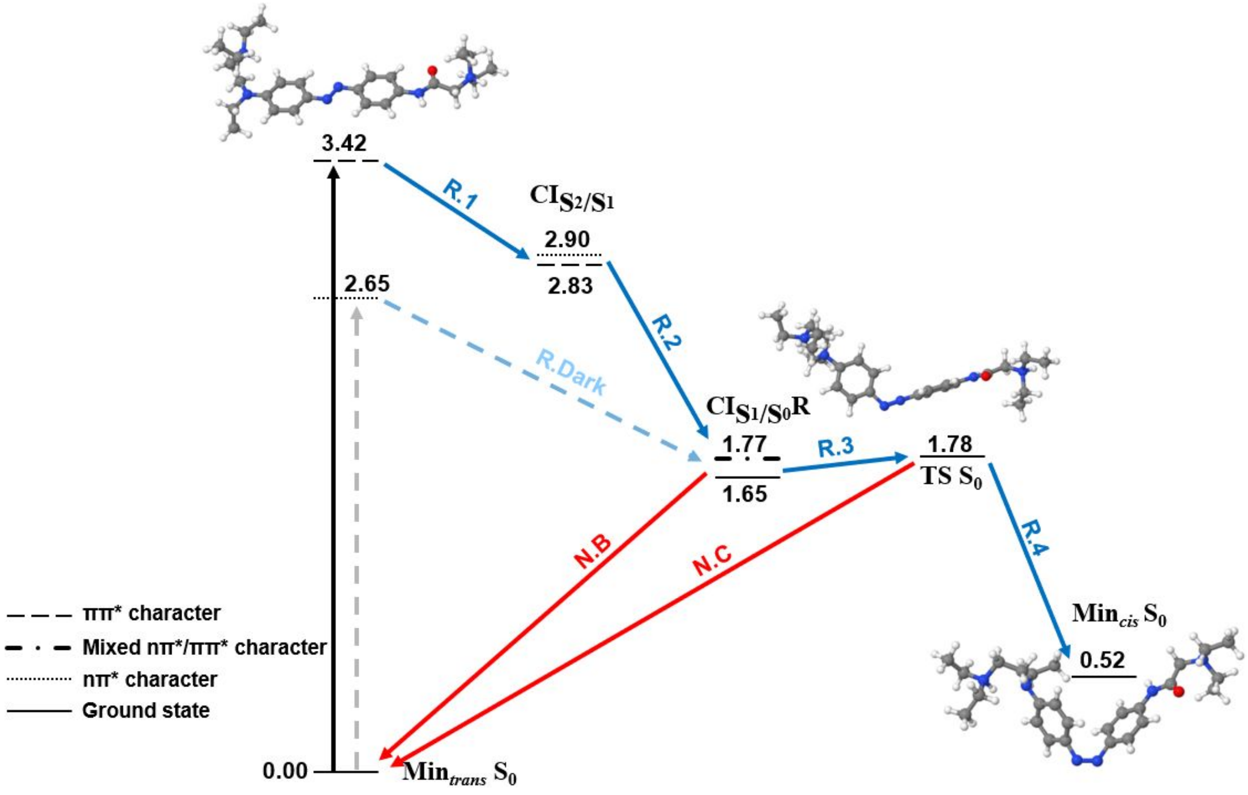

Figure S6. Relaxation pathways of *trans*-$DADH_2^{2+}$ in water at M06-2X/cc-pVDZ. Blue and red arrows indicate reactive (R) and non-reactive pathways (N), respectively. Continuous and dashed arrows represent the bright ππ* and dark nπ* excitations, respectively, and their corresponding relaxation pathways. Energies in eV relative to Min_{trans} S_0.

Table S5. Geometrical parameters of the azo moiety (C-N=N-C dihedral, and C-N=N and N=N-C angles in °) at relevant points of the deactivation pathway of *trans*-$DADH_2^{+2}$ in water at M06-2X/cc-pVDZ.

	Min_{trans} S_0	$CI_{S2/S1}$	$CI_{S1/S0}$	TS S_0	Min_{cis} S_0
**C-N=N-C**	-179.9	-179.2	115.9	93.0	-7.9

**C-N=N**	115.0	112.9	125.8	116.6	122.2
**N=N-C**	114.5	114.0	125.0	179.2	122.5

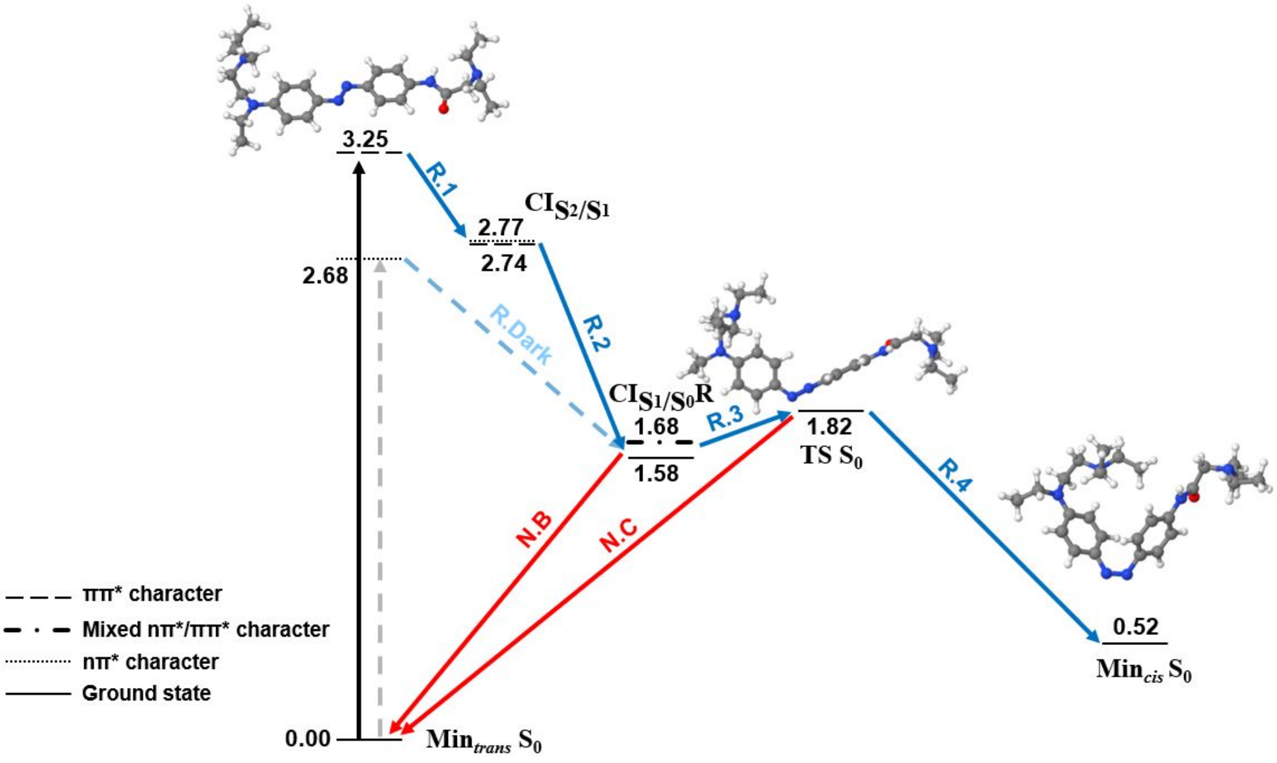

Figure S7. Relaxation pathways of *trans*-DAD in water at M06-2X/cc-pVDZ. Blue and red arrows indicate reactive (R) and non-reactive pathways (N), respectively. Continuous and dashed arrows represent the bright ππ* and dark nπ* excitations, respectively, and their corresponding relaxation pathways. Energies in eV relative to Min$_{trans}$ S_0.

Table S6. Geometrical parameters of the azo moiety (C-N=N-C dihedral, and C-N=N and N=N-C angles in °) at relevant points of the deactivation pathway of *trans*-DAD in water at M06-2X/cc-pVDZ.

	**Min$_{trans}$ S_0**	**CI$_{S2/S1}$**	**CI$_{S1/S0}$**	**TS S_0**	**Min$_{cis}$ S_0**
**C-N=N-C**	179.9	-179.9	116.8	-92.7	8.7

**C-N=N**	115.3	114.7	127.3	117.3	122.5
**N=N-C**	114.5	114.4	127.0	179.9	121.6

The photoisomerization pathways calculated with M06-2X (Figures S3-7, Tables S2-6) estimate larger energy gaps in the Franck-Condon region compared to B3LYP (Figures 3-7, Tables 1-3, 5-6). Furthermore, we did not find the non-reactive nπ* minimum in the M06-2X surfaces in vacuum.

Nevertheless, based on the energetic PES profiles, the general conclusions regarding azobenzene derivation, protonation and solvation are the same as with B3LYP, i.e., derivation, non-protonation and solvation seem to favor the *trans-cis* photoisomerization. These similar results indicate that the partial intramolecular charge transfer character in the S_1 state do not prevent B3LYP from providing a good enough qualitative picture for our purposes.

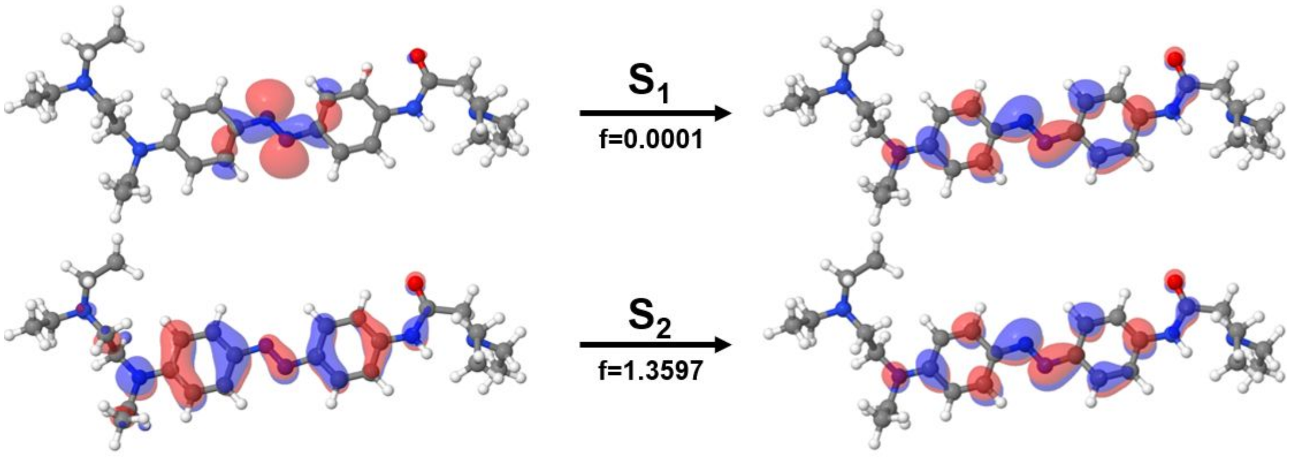

Figure S8. Molecular Orbitals involved in the first (S_1) and second (S_2) excited states of *trans*-DAD in vacuum at the B3LYP/cc-pVDZ level of theory. The oscillator strengths (f) are specified below the arrow.

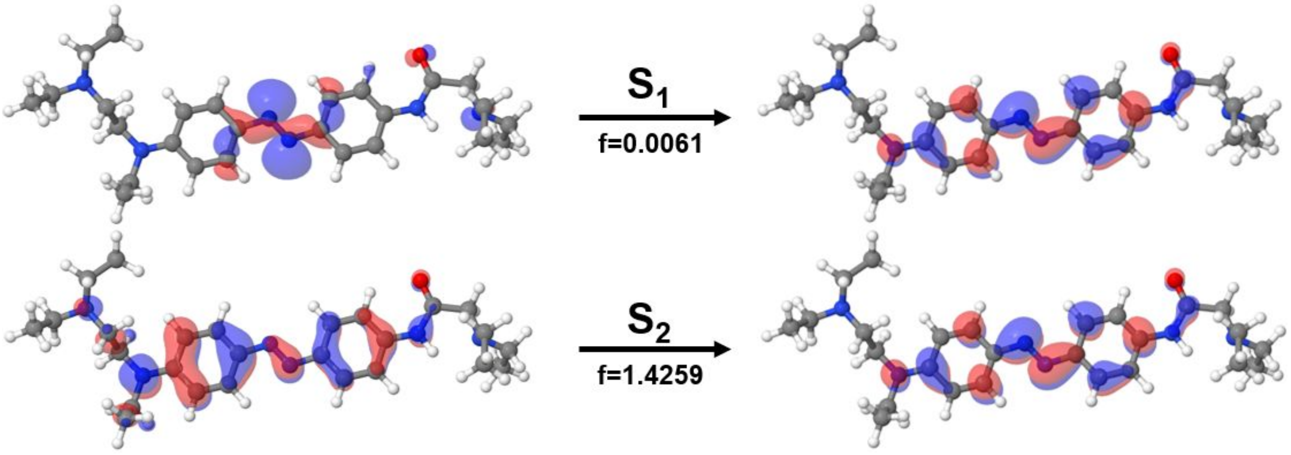

Figure S9. Molecular Orbitals involved in the first (S_1) and second (S_2) excited states of *trans*-DAD in water at the B3LYP/cc-pVDZ level of theory. The oscillator strengths (f) are specified below the arrow.

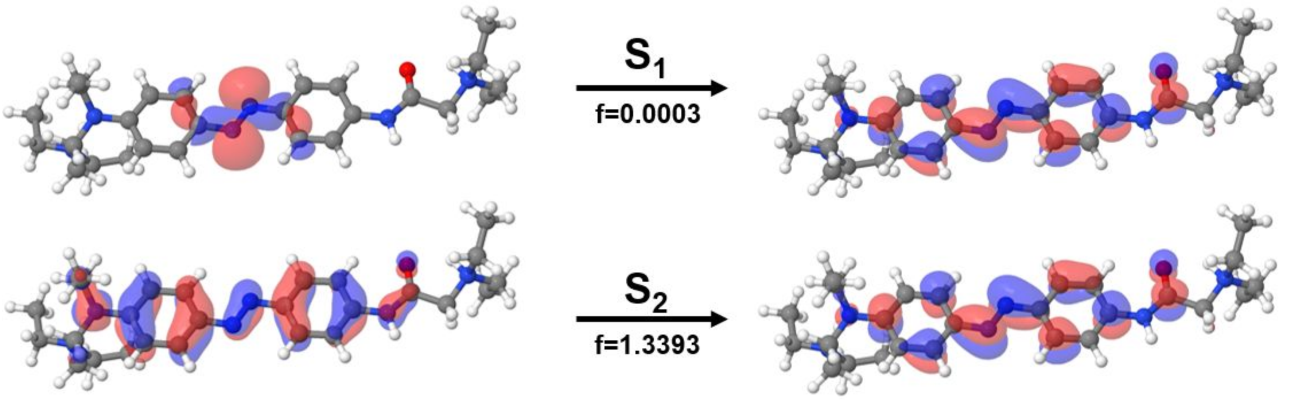

Figure S10. Molecular Orbitals involved in the first (S_1) and second (S_2) excited states of *trans*-$DADH_2^{2+}$ in vacuum at the B3LYP/cc-pVDZ level of theory. The oscillator strengths (f) are specified below the arrow.

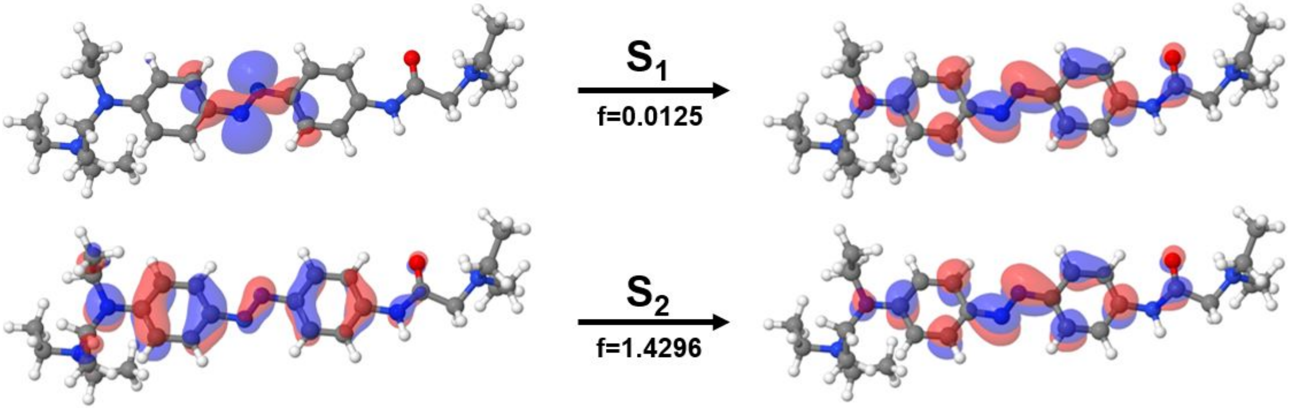

Figure S11. Molecular Orbitals involved in the first (S_1) and second (S_2) excited states of *trans*-$DADH_2^{2+}$ in water at the B3LYP/cc-pVDZ level of theory. The oscillator strengths (f) are specified below the arrow.

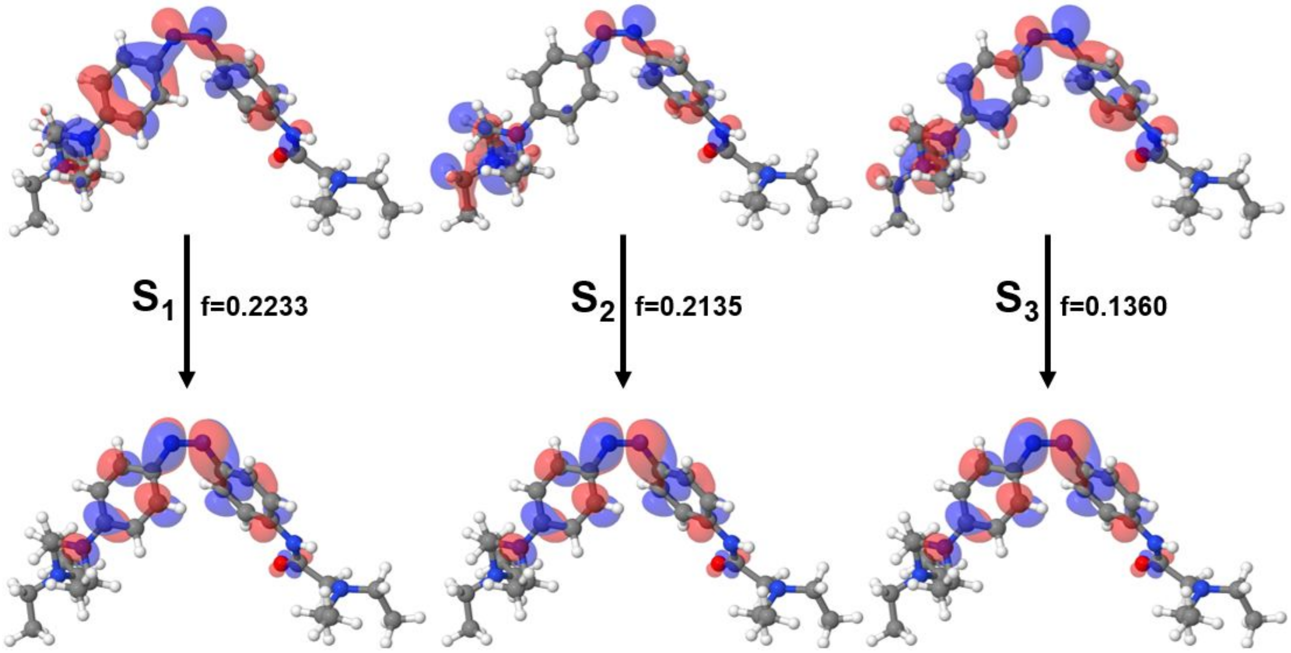

Figure S12. Molecular Orbitals involved in the first (S_1), second (S_2) and third (S_3) excited states of *cis*-DAD in water at the B3LYP/cc-pVDZ level of theory. The oscillator strengths (f) are specified next to the arrow.

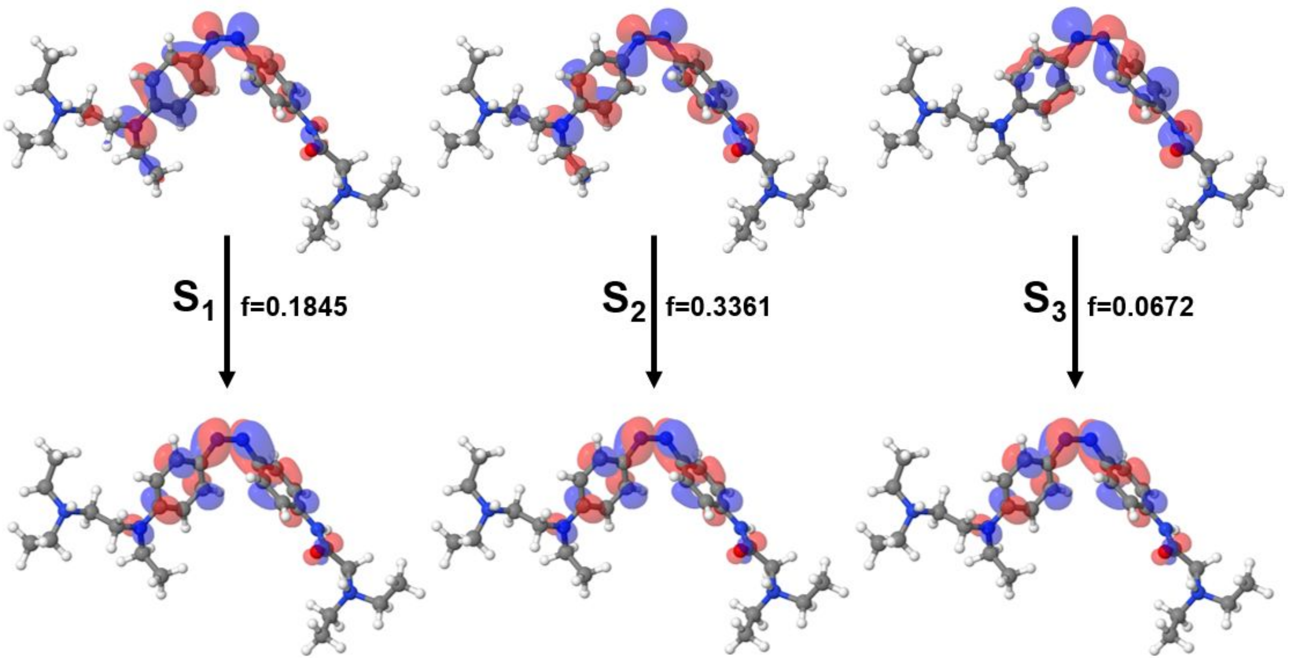

Figure S13. Molecular Orbitals involved in the first (S_1), second (S_2) and third (S_3) excited states of *cis*-$DADH_2^{2+}$ in water at the B3LYP/cc-pVDZ level of theory. The oscillator strengths (f) are specified next to the arrow.

Table S7. Detailed excited states results of $DADH_2^{2+}$ in vacuum at the B3LYP/cc-pVDZ level of theory.

Optimized geometries	Electronic state	Vertical energy (eV)	Adiabatic energy (eV)	Character (weight)	Oscillator strength	Figure orbitals
	S_0	0.00	0.00	-	-	
**Min$_{trans}$ S$_0$**	S_1	2.50	2.50	nπ* (0.69)	0.0003	S10
	S_2	3.29	3.29	ππ* (0.70)	1.3393	
	S_0	0.00	0.24	-	-	
**Min S$_2$ (ππ*)**	S_1	2.43	2.67	nπ* (0.70)	0.0007	S14
	S_2	2.78	3.02	ππ* (0.71)	1.4417	
**CI$_{S2/S1}$**	S_0	0.00	0.38	-	-	S15

	S_1	2.76	3.14	ππ* (0.71)	1.2397	
	S_2	2.82	3.20	nπ* (0.56)<br>nπ* (0.41)	0.0008	
**Min S_1 (nπ*)**	S_0	0.00	0.77	-	-	S16
	S_1	1.11	1.88	nπ*(0.70)	0.0004	
**$CI_{S1/S0}$ NR**	S_0	0.00	2.10	-	-	S17
	S_1	0.15	2.25	nπ*(-0.71)	0.0000	
**$CI_{S1/S0}$ R**	S_0	0.00	1.88	-	-	S18
	S_1	0.07	1.95	Mixed nπ*/ππ* (-1.26)	0.0006	

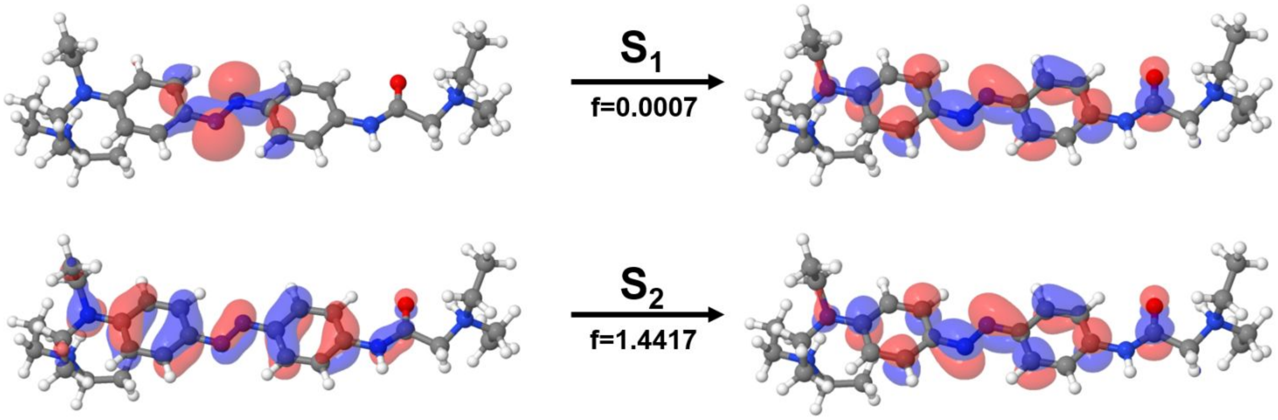

Figure S14. Molecular Orbitals involved in the first (S_1) and second (S_2) excited states of the S_2 (ππ*) minimum of $DADH_2^{2+}$ in vacuum at the B3LYP/cc-pVDZ level of theory. The oscillator strengths (f) are specified below the arrow.

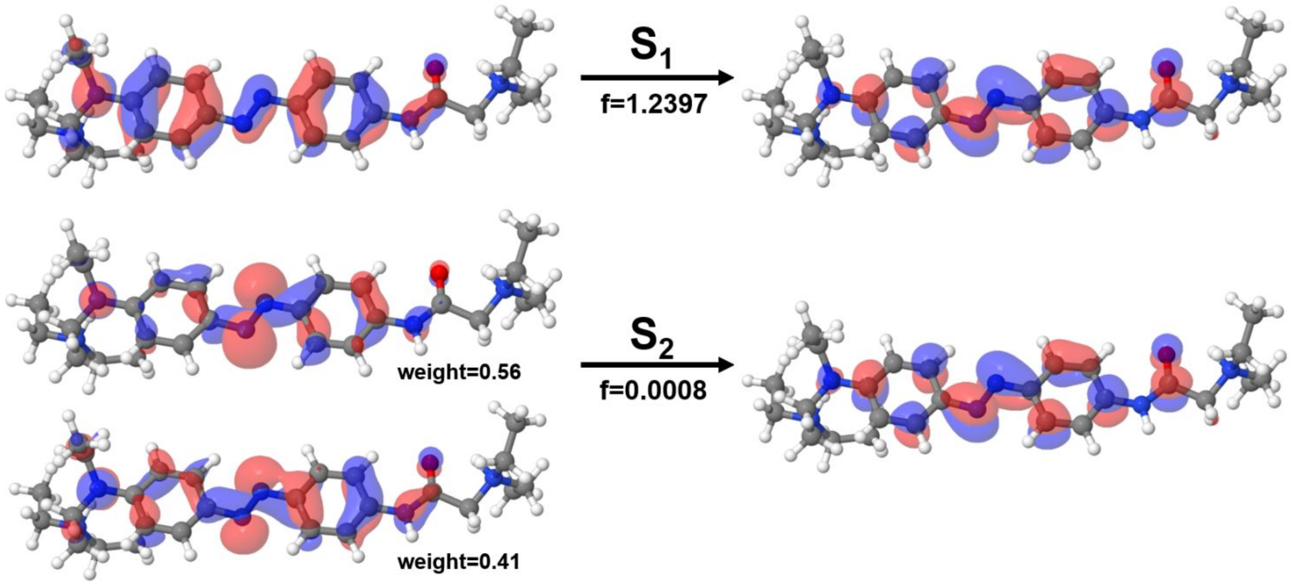

Figure S15. Molecular Orbitals involved in the first (S_1) and second (S_2) excited states of the $DADH_2^{2+}$ $CI_{S2/S1}$ in vacuum at the B3LYP/cc-pVDZ level of theory. The oscillator strengths (f) are specified below the arrow.

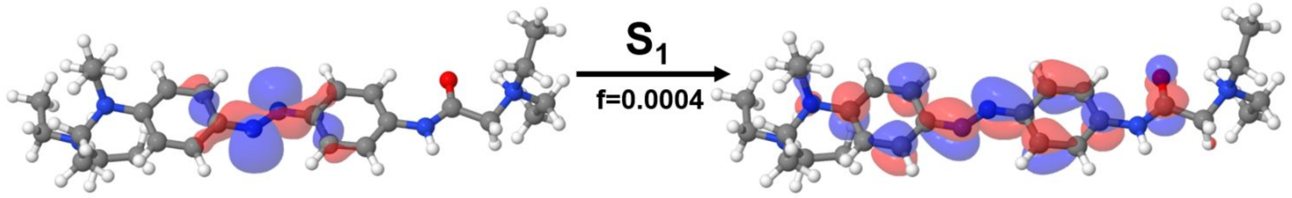

Figure S16. Molecular Orbitals involved in the first (S_1) excited state of the S_1 (nπ*) minimum of $DADH_2^{2+}$ in vacuum at the B3LYP/cc-pVDZ level of theory. The oscillator strength (f) is specified below the arrow.

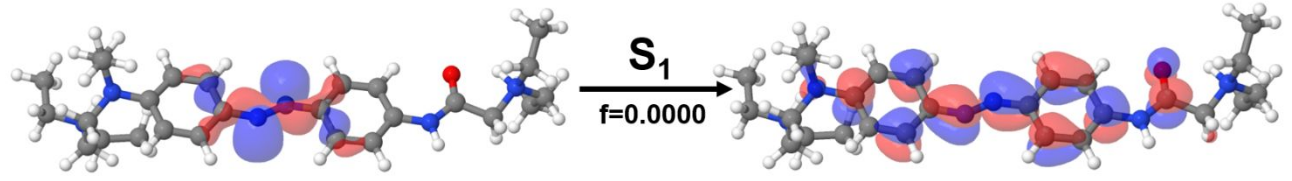

Figure S17. Molecular Orbitals involved in the first (S_1) excited state of the non-reactive $DADH_2^{2+}$ $CI_{S1/S0}$ in vacuum at the B3LYP/cc-pVDZ level of theory. The oscillator strength (f) is specified below the arrow.

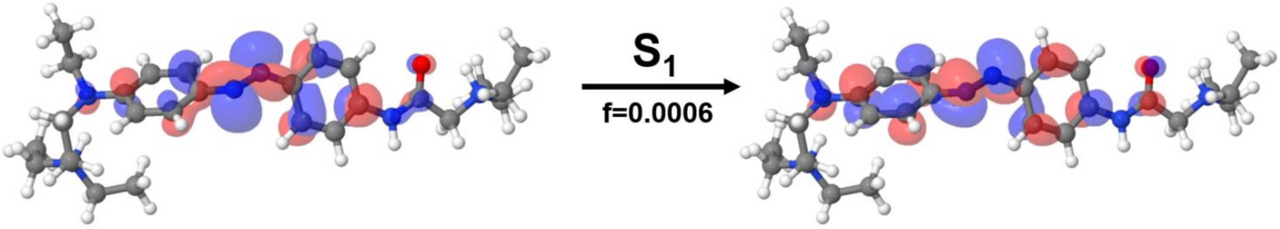

Figure S18. Molecular Orbitals involved in the first (S_1) excited state of the reactive $DADH_2^{2+}$ $CI_{S1/S0}$ in vacuum at the B3LYP/cc-pVDZ level of theory. The oscillator strength (f) is specified below the arrow.

Geometry	Energy (eV)

Min S_1 (nπ*)

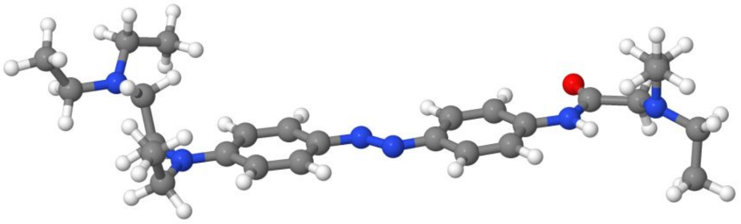

0.0

Intermediate 1

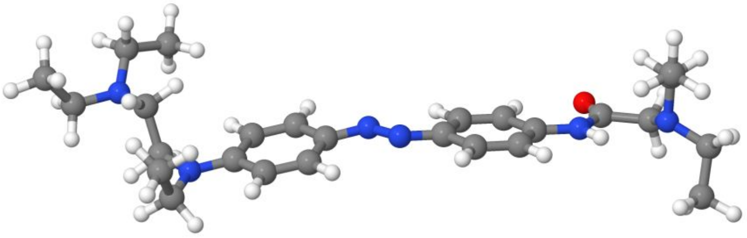

0.03

Intermediate 2

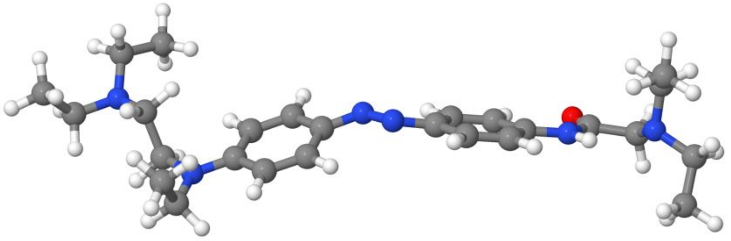

0.07

Intermediate 3

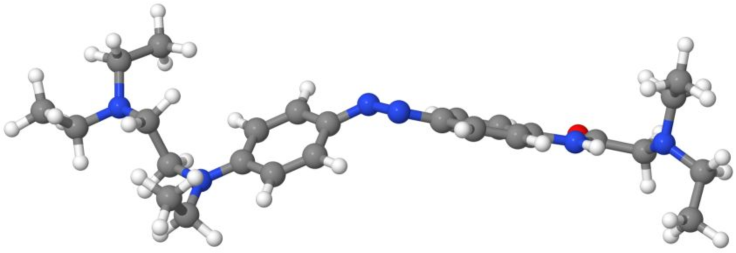

0.09

Intermediate 4

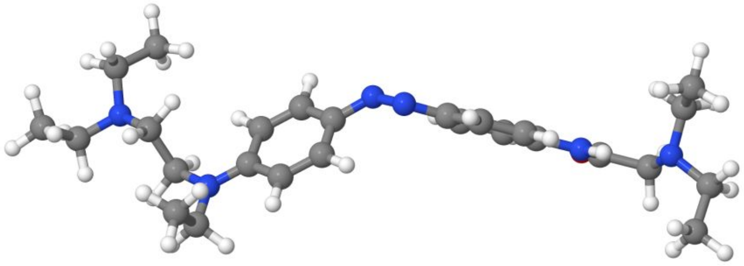

0.10

Reactive S_1/S_0 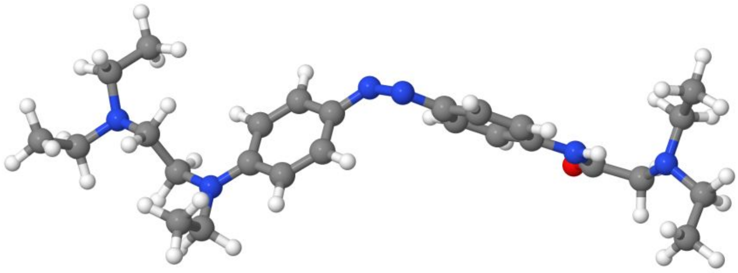 0.18

Figure S19. Geometries of the NEB calculation between the S_1 minimum and the reactive S_1/S_0 crossing point of DAD in vacuum at the B3LYP/cc-pVDZ level of theory (R.S path in Figure 5). Energies in eV with respect to the S_1 minimum.

The R.S path of DAD in vacuum (Figure 5, main text) was further explored by a NEB calculation in which we requested 4 intermediate structures in between the non-reactive nπ* minimum in the S_1 surface and the reactive S_1/S_0 crossing point. According to this calculation, the highest energy structure along the R.S path corresponds to the reactive S_1/S_0 crossing point, which is 0.18 eV more energetic than the S_1 minimum. This low energy difference probably makes the R.S path more accessible than the non-reactive and more energetic N.A.2 path (Figure 5, main text).

References

(1) Pracht, P.; Bohle, F.; Grimme, S. Automated Exploration of the Low-Energy Chemical Space with Fast Quantum Chemical Methods. *Phys Chem Chem Phys* **2020**, *22* (14), 7169–7192. https://doi.org/10.1039/C9CP06869D.

(2) Genheden, S.; Ryde, U. The MM/PBSA and MM/GBSA Methods to Estimate Ligand-Binding Affinities. *Expert Opin Drug Discov* **2015**, *10* (5), 449–461. https://doi.org/10.1517/17460441.2015.1032936.

(3) Bannwarth, C.; Ehlert, S.; Grimme, S. GFN2-XTB—An Accurate and Broadly Parametrized Self-Consistent Tight-Binding Quantum Chemical Method with Multipole Electrostatics and Density-Dependent Dispersion Contributions. *J Chem Theory Comput* **2019**, *15* (3), 1652–1671. https://doi.org/10.1021/acs.jctc.8b01176.

(4) Spicher, S.; Grimme, S. Robust Atomistic Modeling of Materials, Organometallic, and Biochemical Systems. *Angew Chem Int Ed* **2020**, *59* (36), 15665–15673. https://doi.org/10.1002/anie.202004239.

(5) Li, Y.; Wu, H. A Clustering Method Based on K-Means Algorithm. *Phys Procedia* **2012**, *25*, 1104–1109. https://doi.org/10.1016/j.phpro.2012.03.206.

(6) Roe, D. R.; Cheatham, T. E. PTRAJ and CPPTRAJ: Software for Processing and Analysis of Molecular Dynamics Trajectory Data. *J Chem Theory Comput* **2013**, *9* (7), 3084–3095. https://doi.org/10.1021/ct400341p.

(7) Tomasi, J.; Mennucci, B.; Cancès, E. The IEF Version of the PCM Solvation Method: An Overview of a New Method Addressed to Study Molecular Solutes at the QM Ab Initio Level. *J Mol Struct-THEOCHEM* **1999**, *464* (1–3), 211–226. https://doi.org/10.1016/S0166-1280(98)00553-3.

(8) Miertuš, S.; Scrocco, E.; Tomasi, J. Electrostatic Interaction of a Solute with a Continuum. A Direct Utilizaion of AB Initio Molecular Potentials for the Prevision of Solvent Effects. *Chem Phys* **1981**, *55* (1), 117–129. https://doi.org/10.1016/0301-0104(81)85090-2.

(9) Mennucci, B.; Cammi, R.; Tomasi, J. Excited States and Solvatochromic Shifts within a Nonequilibrium Solvation Approach: A New Formulation of the Integral Equation Formalism Method at the Self-Consistent Field, Configuration Interaction, and Multiconfiguration Self-Consistent Field Level. *J Chem Phys* **1998**, *109* (7), 2798–2807. https://doi.org/10.1063/1.476878.

(10) Stephens, P. J.; Devlin, F. J.; Chabalowski, C. F.; Frisch, M. J. Ab Initio Calculation of Vibrational Absorption and Circular Dichroism Spectra Using Density Functional Force Fields. *J Phys Chem* **1994**, *98* (45), 11623–11627. https://doi.org/10.1021/j100096a001.

(11) Vosko, S. H.; Wilk, L.; Nusair, M. Accurate Spin-Dependent Electron Liquid Correlation Energies for Local Spin Density Calculations: A Critical Analysis. *Can J Phys* **1980**, *58* (8), 1200–1211. https://doi.org/10.1139/p80-159.

(12) Lee, C.; Yang, W.; Parr, R. G. Development of the Colle-Salvetti Correlation-Energy Formula into a Functional of the Electron Density. *Phys Rev B* **1988**, *37* (2), 785–789. https://doi.org/10.1103/PhysRevB.37.785.

(13) Becke, A. D. Density-Functional Thermochemistry. III. The Role of Exact Exchange. *J Chem Phys* **1993**, *98* (7), 5648–5652. https://doi.org/10.1063/1.464913.

(14) Yanai, T.; Tew, D. P.; Handy, N. C. A New Hybrid Exchange–Correlation Functional Using the Coulomb-Attenuating Method (CAM-B3LYP). *Chem Phys Lett* **2004**, *393* (1–3), 51–57. https://doi.org/10.1016/j.cplett.2004.06.011.

(15) Zhao, Y.; Truhlar, D. G. The M06 Suite of Density Functionals for Main Group Thermochemistry, Thermochemical Kinetics, Noncovalent Interactions, Excited States, and

Transition Elements: Two New Functionals and Systematic Testing of Four M06-Class Functionals and 12 Other Functionals. *Theor Chem Acc* **2008**, *120* (1–3), 215–241. https://doi.org/10.1007/s00214-007-0310-x.

(16) Dunning, T. H. Gaussian Basis Sets for Use in Correlated Molecular Calculations. I. The Atoms Boron through Neon and Hydrogen. *J Chem Phys* **1989**, *90* (2), 1007–1023. https://doi.org/10.1063/1.456153.

(17) Frisch, M. J.; Trucks, G. W.; Schlegel, H. B.; Scuseria, G. E.; Robb, M. A.; Cheeseman, J. R.; Scalmani, G.; Barone, V.; Petersson, G. A.; Nakatsuji, H.; et al. Gaussian~16 Revision C.01. 2016.

(18) Laurent, A. D.; Jacquemin, D. TD-DFT Benchmarks: A Review. *Int J Quantum Chem* **2013**, *113* (17), 2019–2039. https://doi.org/10.1002/qua.24438.

(19) O'Boyle, N. M.; Banck, M.; James, C. A.; Morley, C.; Vandermeersch, T.; Hutchison, G. R. Open Babel: An Open Chemical Toolbox. *J Cheminform* **2011**, *3* (1), 33. https://doi.org/10.1186/1758-2946-3-33.

(20) Slater, J. C. A Simplification of the Hartree-Fock Method. *Phys Rev* **1951**, *81* (3), 385–390. https://doi.org/10.1103/PhysRev.81.385.

(21) Hehre, W. J.; Ditchfield, R.; Pople, J. A. Self—Consistent Molecular Orbital Methods. XII. Further Extensions of Gaussian—Type Basis Sets for Use in Molecular Orbital Studies of Organic Molecules. *J Chem Phys* **1972**, *56* (5), 2257–2261. https://doi.org/10.1063/1.1677527.

(22) Hariharan, P. C.; Pople, J. A. The Influence of Polarization Functions on Molecular Orbital Hydrogenation Energies. *Theor Chim Acta* **1973**, *28* (3), 213–222. https://doi.org/10.1007/BF00533485.

(23) Ditchfield, R.; Hehre, W. J.; Pople, J. A. Self-Consistent Molecular-Orbital Methods. IX. An Extended Gaussian-Type Basis for Molecular-Orbital Studies of Organic Molecules. *J Chem Phys* **1971**, *54* (2), 724–728. https://doi.org/10.1063/1.1674902.

(24) Case, D. A.; Belfon, K.; Ben-Shalom, I. Y.; Brozell, S. R.; Cerutti, D. S.; Cheatham, T. E.; III; Cruzeiro, V. W. D.; Darden, T. A.; Duke, R. E.; G. Giambasu, M. K. G.; et al. AMBER 2020. 2020.

(25) Mark, P.; Nilsson, L. Structure and Dynamics of the TIP3P, SPC, and SPC/E Water Models at 298 K. *J Phys Chem A* **2001**, *105* (43), 9954–9960. https://doi.org/10.1021/jp003020w.

(26) He, X.; Man, V. H.; Yang, W.; Lee, T.-S.; Wang, J. A Fast and High-Quality Charge Model for the next Generation General AMBER Force Field. *J Chem Phys* **2020**, *153* (11). https://doi.org/10.1063/5.0019056.

(27) Davidchack, R. L.; Handel, R.; Tretyakov, M. V. Langevin Thermostat for Rigid Body Dynamics. *J Chem Phys* **2009**, *130* (23). https://doi.org/10.1063/1.3149788.

(28) Berendsen, H. J. C.; Postma, J. P. M.; van Gunsteren, W. F.; DiNola, A.; Haak, J. R. Molecular Dynamics with Coupling to an External Bath. *J Chem Phys* **1984**, *81* (8), 3684–3690. https://doi.org/10.1063/1.448118.

(29) Ryckaert, J.-P.; Ciccotti, G.; Berendsen, H. J. C. Numerical Integration of the Cartesian Equations of Motion of a System with Constraints: Molecular Dynamics of n-Alkanes. *J Comput Phys* **1977**, *23* (3), 327–341. https://doi.org/10.1016/0021-9991(77)90098-5.

(30) McCullagh, M.; Franco, I.; Ratner, M. A.; Schatz, G. C. DNA-Based Optomechanical Molecular Motor. *J Am Chem Soc* **2011**, *133* (10), 3452–3459. https://doi.org/10.1021/ja109071a.

(31) Essmann, U.; Perera, L.; Berkowitz, M. L.; Darden, T.; Lee, H.; Pedersen, L. G. A Smooth Particle Mesh Ewald Method. *J Chem Phys* **1995**, *103* (19), 8577–8593. https://doi.org/10.1063/1.470117.

(32) Cárdenas, G.; Lucia-Tamudo, J.; Mateo-delaFuente, H.; Palmisano, V. F.; Anguita-Ortiz, N.; Ruano, L.; Pérez-Barcia, Á.; Díaz-Tendero, S.; Mandado, M.; Nogueira, J. J. MoBioTools%: A Toolkit to Setup Quantum Mechanics/Molecular Mechanics Calculations. *J Comput Chem* **2023**, *44* (4), 516–533. https://doi.org/10.1002/jcc.27018.

(33) Mai, S.; Marquetand, P.; González, L. Nonadiabatic Dynamics: The SHARC Approach. *Wiley Interdiscip Rev Comput Mol Sci* **2018**, *8* (6). https://doi.org/10.1002/wcms.1370.

(34) Mai, S.; Richter, M.; Heindl, M.; Menger, M. F. S. J.; Atkins, A. J.; Ruckenbauer, M.; Plasser, F.; Ibele, L. M.; Kropf, S.; Oppel, M.; et al. SHARC2. 1: Surface Hopping Including Arbitrary Couplings–Program Package for Non-Adiabatic Dynamics. *sharc-md. org* **2019**, *30*.

(35) Neese, F. Software Update: The ORCA Program System—Version 5.0. *Wiley Interdiscip Rev Comput Mol Sci* **2022**, *12* (5). https://doi.org/10.1002/wcms.1606.

(36) Isokuortti, J.; Griebenow, T.; Glasenapp, J.-S. von; Raeker, T.; Filatov, M. A.; Laaksonen, T.; Herges, R.; Durandin, N. A. Triplet Sensitization Enables Bidirectional Isomerization of Diazocine with 130 Nm Redshift in Excitation Wavelengths. *Chem Sci* **2023**, *14* (34), 9161–9166. https://doi.org/10.1039/D3SC02681G.

(37) Casellas, J.; Bearpark, M. J.; Reguero, M. Excited-State Decay in the Photoisomerisation of Azobenzene: A New Balance between Mechanisms. *ChemPhysChem* **2016**, *17* (19), 3068–3079. https://doi.org/10.1002/cphc.201600502.

(38) Conti, I.; Garavelli, M.; Orlandi, G. The Different Photoisomerization Efficiency of Azobenzene in the Lowest Nπ* and Ππ* Singlets: The Role of a Phantom State. *J Am Chem Soc* 2008, *130* (15), 5216–5230. https://doi.org/10.1021/ja710275e.

(39) Jmol: An Open-Source Java Viewer for Chemical Structures in 3D. Http://Www.Jmol.Org/.
